# Multi-omics data reveals the important role of glycerophospholipid metabolism in the crosstalk between gut and brain in depression

**DOI:** 10.1186/s12967-023-03942-w

**Published:** 2023-02-07

**Authors:** Jing Xie, Qi Zhong, Wen-tao Wu, Jian-jun Chen

**Affiliations:** 1 Chongqing Emergency Medical Center, Central Hospital of Chongqing University, Chongqing, 400010 China; 2 Institute of Life Sciences, Chongqing Medical University, 1 Yixueyuan Road, Yuzhong District, Chongqing, 400016 China

**Keywords:** Depression, Gut microbiota, Glycerophospholipids, Neurotransmitter, Lachnospiraceae, GABA

## Abstract

**Background:**

Gut microbiota plays a critical role in the onset and development of depression, but the underlying molecular mechanisms are unclear. This study was conducted to observe the characteristics of gut microbiota, lipid metabolism and neurotransmitters in Gut-Liver-Brain axis in depressed mice (DM), and identify some novel perceptions on relationships between gut microbiota and depression.

**Methods:**

A mouse model of depression was built used chronic unpredictable mild stress (CUMS). Fecal samples (measuring gut microbiota compositions, microbial genes and lipid metabolites), liver samples (measuring lipid metabolites), and hippocampus (measuring neurotransmitters) were collected. Both univariate and multivariate statistical analyses were used to identify the differential gut microbiota, metabolic signatures and neurotransmitters in DM.

**Results:**

There were significant differences on both microbial and metabolic signatures between DM and control mice (CM): 71 significantly changed operational taxonomic units (OTUs) (60.56% belonged to phylum Firmicutes) and 405 differential lipid metabolites (51.11% belonged to Glycerophospholipid (GP) metabolism) were identified. Functional analysis showed that depressive-like behaviors (DLB)-related differential microbial genes were mainly enriched in GP metabolism. Weighted correlation network analysis (WGCNA) showed that DLB-related differential metabolites mainly belonged to GPs. Meanwhile, seven differential neurotransmitters were identified. Comprehensive analysis found that Lachnospiraceae and gamma-aminobutyric acid (GABA) were significantly correlated with 94.20% and 53.14% differential GPs, respectively, and GABA was significantly correlated with three main DLB phenotypes.

**Conclusion:**

Our results provided novel perceptions on the role of Gut-Liver-Brain axis in the onset of depression, and showed that GP metabolism might be the bridge between gut microbiota and depression. “Lachnospiraceae-GP metabolism-GABA” held the promise as a potential way between gut microbiota and brain functions in DM.

**Supplementary Information:**

The online version contains supplementary material available at 10.1186/s12967-023-03942-w.

## Introduction

Depression, one of the leading causes of disability, is a common mental disease in clinical practice [1]. It is estimated that about 300 million people living with this disorder [2]. The main symptoms of depression include anhedonia, loss of appetite, sleep disorders, depressed mood, and sense of worthlessness [3]. This disease brings a massive economic burden to individuals and society. Moreover, if not timely and effectively treated, depression patients are likely to experience suicidal ideation or suicide attempts [4]. Our previous study showed that the continuously decreased level of alpha 1-antitrypsin in depression patients might result in the appearance of suicidal ideation [5]. However, up to now, the pathogenesis of depression is still not fully elucidated, and the widely accepted diagnostic biomarkers are still not available. This phenomenon suggests that it may be meaningful to explore the pathogenesis of depression from new aspects.

Gut microbiota plays an essential role in maintaining the health of host, and the crosstalk of gut and brain has become a research hotspot [6–8]. Mounting evidence showed that there were close relationships between gut microbiota and central nervous system (CNS) functions [9, 10]. In the recent decade, researches on the role of gut microbiota in the onset and development of depression have increased significantly. Our previous animal models and clinical studies showed that both depressed mice (DM) and patients had the significantly changed gut microbiota compositions [11–14]. Meanwhile, we found that the control mice (CM) showed depressive-like behavior after fecal transplantation (fecal samples were obtained from depression patients) [14]. Recent evidence indicates that the metabolic products of gut microbiota can regulate the brain functions of host through “microbiota-gut-brain” axis [15, 16]. However, the underlying molecular mechanisms between brain functions and gut microbiota are still not completely elucidated.

Evidence has shown that the lipid metabolism of host can be affected by gut microbiota [17]. Interestingly, our previous studies have identified some significantly changed lipid metabolism-related metabolites in patients with depression [18–20] Meanwhile, using two mouse models of depression, we provided some evidence that the lipid metabolism in liver was significantly affected by gut microbiota [21]. Glycerophospholipids (GP) are the major structural lipid components of eukaryote cellular membranes and participate in many cellular processes. Our previous study reported that GP metabolism in hippocampus of post-stroke depression rats was significantly disordered [22]. Meanwhile, using germ-free mice, we built a mouse model of depression by fecal microbiota transplantation from patients with depression and healthy controls; and we found that GP metabolism was significantly changed between DM and CM [21]. These findings indicated that GP metabolism might have an important role in the crosstalk of gut and brain.

In addition, neurotransmitters, as the chemical messengers in CNS, have close relationships with depression. Using chronic restraint stress (CRS)-induced depression model, we found three significantly decreased neurotransmitters in the hypothalamus of DM [13]. Using chronic unpredictable mild stress (CUMS)-induced depression model, we identified several differential neurotransmitters in GABAergic and Catecholaminergic pathways in prefrontal cortex of DM [23]. Della et al. reported that the current evidence strongly supported the presence of a deficiency of GABA system in depression, which appeared to be restored after treatment [24]. Moreover, we found that neurotransmitters in plasma held the promise as the diagnostic biomarkers for patients with depression, such as GABA and kynurenine [25]. Based on the above-mentioned findings, this study was performed to further observe the characteristics of gut microbiota, lipid metabolism and neurotransmitters in Gut-Liver-Brain axis in depressed mice. Our results could provide some novel perceptions on the relationships between gut microbiota and depression.

## Methods

### Experimental animal

C57BL/6 mice (~ 20 g, adult male) were provided by the Laboratory Animal Center of Chongqing Medical University. The mice were housed in isolation under the standard conditions: humidity of 52 ± 2%; temperature of 22 ± 1 °C; 12 h: 12 h light: dark cycle; with ad libitum access to water and food. All the behavioral tests were conducted in strict accordance with National Institutes of Health (NIH) guidelines and approved by the Ethics Committee of Chongqing Medical University (Approval No. 20170301).

### Depression model building

A mouse model of depression was built here used CUMS. After one week of adaption, a computer-generated number was applied to do randomization group. The mice in the experimental group and control group were matched for age, body weight (BW) and sucrose preference (SPF). The mice in the experimental group were exposed to CUMS for four weeks. The CUMS procedure here was mainly performed in accordance with the procedures in our previous studies (Additional File 1) [26, 27]. Briefly, each mouse in the experimental group received one stressor per day, such as cages tilted and wet cages. The same stressor was not used for two consecutive days. At last, the food and water were deprived for 24 h before conducting behavioral experiments. The mice in the control group were free access to water and food without disturbance. To avoid the potential olfactory or acoustic communications between the two groups, the mice in the control groups were far away from the mice in the experimental group.

### Behavioral tests

All the tests were exactly performed in accordance with the procedures in our previous studies (Additional File 1) [13, 26, 27]. The behavioral tests in this study included open field test (OFT), forced swim test (FST) and sucrose preference test (SPT). The related indicators assessing depressive-like behaviors (DLB) were measured. Briefly, after the CUMS procedure, the total distance, distance in center area (CD), time in center area (CT) from the OFT were measured; the immobility time (IT) from the FST was measured; and the SPF from the SPT was measured before and after the CUMS procedure. Meanwhile, the BW was also measured before and after the CUMS procedure. Data from the last five minutes in both OFT and FST was collected, and data from the 24 h in SPT was collected.

### Gut microbiota compositions, lipids and neurotransmitters detection

The procedures of 16S rRNA gene sequence analysis and metagenomic analysis of fecal samples were exactly according to our previous studies (Additional File 1) [13, 14, 28, 29]. Meanwhile, the lipidomics analyses of fecal sample were performed using liquid chromatography-mass spectrometry (LC-MS), and the procedure was also exactly according to our previous studies (Additional File 1) [13, 14, 29]. The lipidomics analyses of liver sample were also performed using LC-MS, and the procedure was described in Additional File 1. In addition, the procedure of neurotransmitters detection in the hippocampus of mice was also exactly according to our previous study (Additional File 1) [13, 29]. The neurotransmitters in GABAergic and Catecholaminergic pathways were detected. Considering our previous findings about the disordered functions of hippocampus in depression [14, 29], only the hippocampal area of the brain was studied in this study.

### Statistical analysis

Firstly, the alpha diversity and beta diversity between DM and CM were assessed using two parameters (Simpson and Shannon) and principal coordinate analysis (PCoA), respectively. Secondly, we used linear discriminant analysis Effective Size (LEfSe) to identify the differential OTUs between the two groups. Thirdly, the Pearson correlation method was used to find out the differential microbial genes significantly correlated with DLB, and then the functional analysis was applied to explore the metabolic pathways that these differential genes were significantly involved in. Fourthly, the orthogonal partial least squares discriminant analysis (OPLS-DA) method was applied to identify the differential lipid metabolites in fecal and liver samples, and then MetaboAnalyst 5.0 was applied to analyze the pathways that these differential metabolites were significantly involved in. Fifthly, the weighted correlation network analysis (WGCNA) was applied to find out the pivotal DLB-related metabolic modules [30, 31]. Finally, comprehensive analysis of these data was performed to identify the potential pathways in Gut-Liver-Brain axis in depression. All statistical analyses were conducted using SPSS 19.0, R software 3.6 and SIMCA 14.1.

## Results

### Behavioral characteristics in DM

**Fig. 1:**
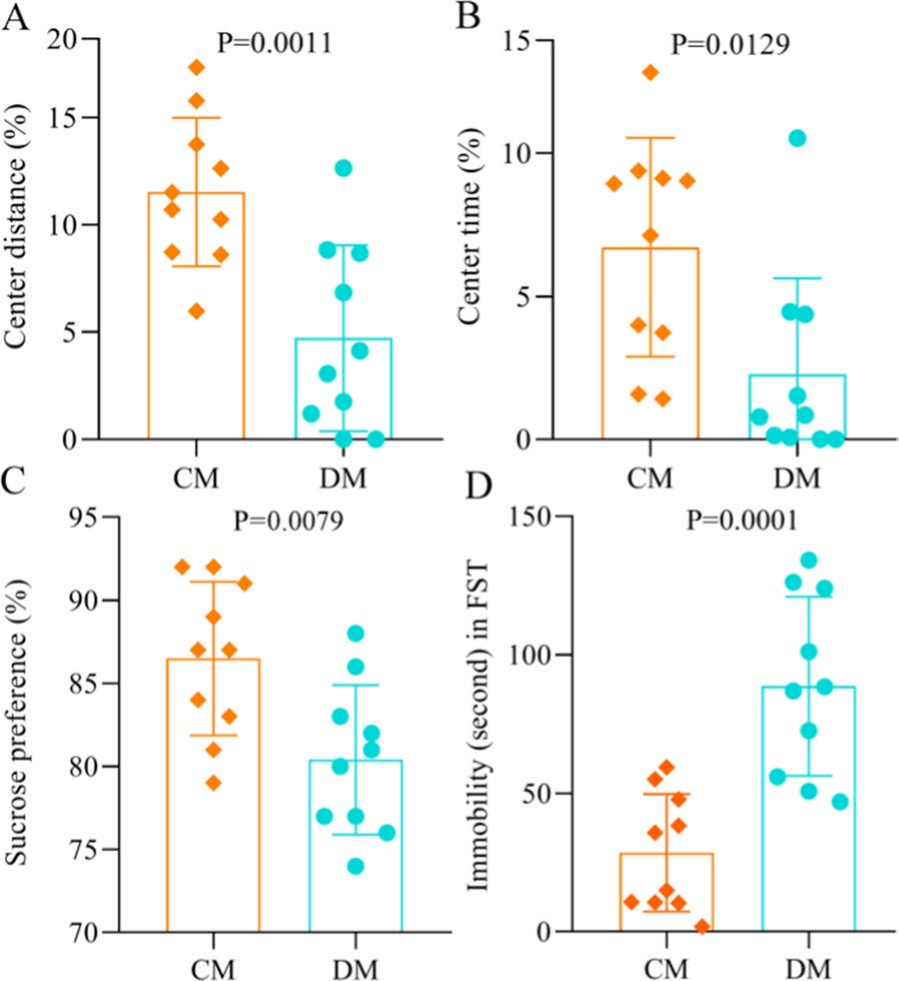
Behavioral phenotypes of depressed mice and control mice. **A**, **B** DM had the significantly decreased center distance (%) **A** and center time (%) **B** in open field test compared to CM; **C** DM had the significantly decreased sucrose preference (%) in sucrose preference test compared to CM; **D** DM had the significantly increased immobility time in forced swimming test compared to CM. *DM* depressed mice, n = 10, *CM* control mice, n = 10

In the OFT, the similar total distance (p = 0.33) between the two groups indicated that the DM and CM had the similar motor functions. Compared to the CM, the DM had the significantly lower CD (p = 0.0011, Fig. 1A) and CT (p = 0.0129, Fig. 1B). The results of SPT showed that there was a significant difference on SPF after experiment between the two groups (p = 0.0079, Fig. 1C). In the FST, the IT was significantly increased in the DM than in the CM (p = 0.0001, Fig. 1D). In addition, the two groups had the similar BW (p = 0.1010) after experiment. These results showed that the mice in the experiment group displayed DLB after four weeks of CUMS. The data of behavioral tests was described in Additional File 1.

### Gut microbiome alterations in DM

**Fig. 2:**
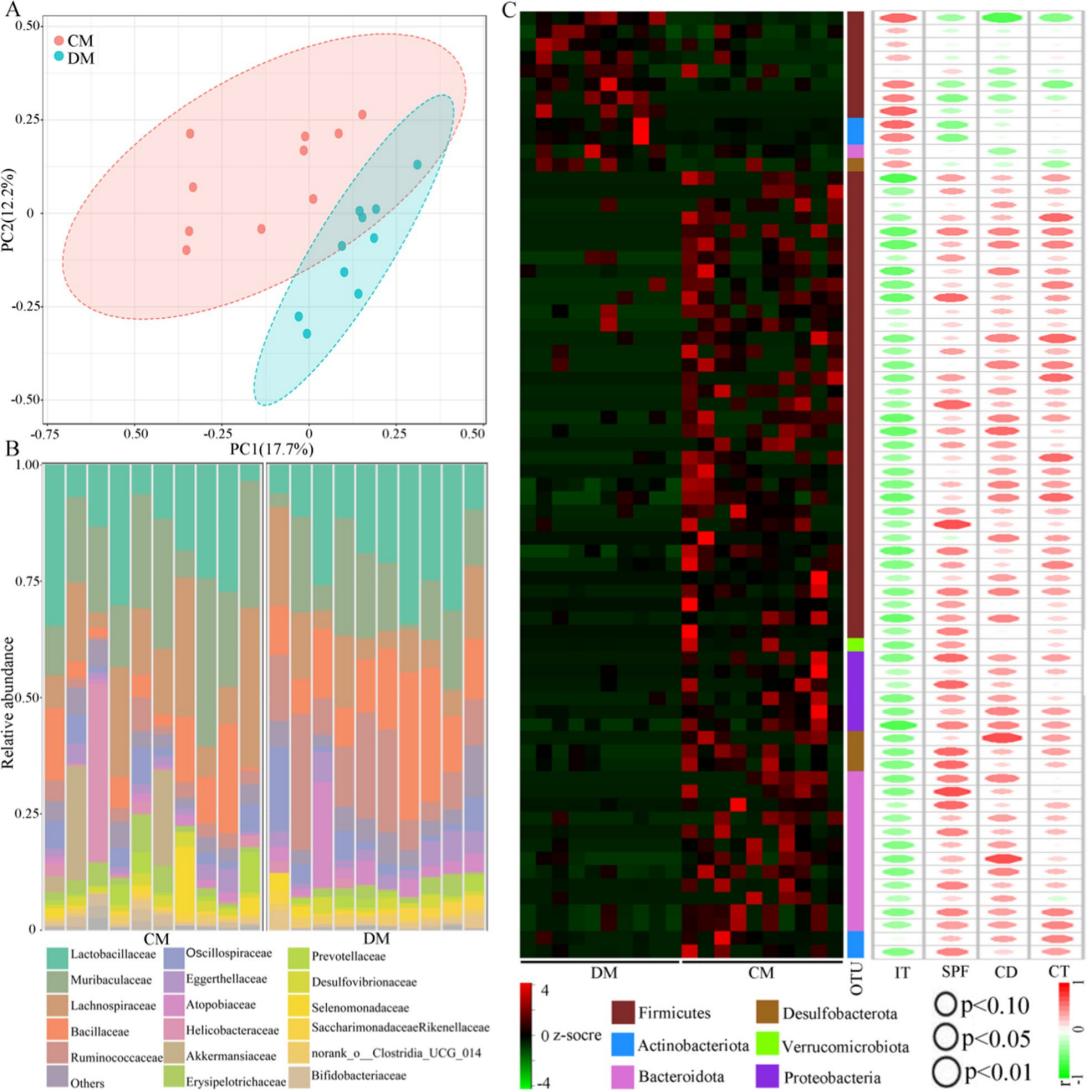
Differences of gut microbiota compositions in depressed mice vs. control mice. **A** There were obvious differences of gut microbiota compositions between the two groups; **B** Relative abundances of OTUs in both groups assigned at the Family level; **C** The heat-map was built using the normalized abundance score for each differential OTU, and all OTUs were classified at phylum level. The r and p values from Pearson correlation method were represented by the color and size of each ellipse in scatter plot, respectively. *DM* depressed mice, n = 10, *CM* control mice, n = 10. *IT* immobility time, *CD* center distance, *SPF* sucrose preference, *CT* center time

The results of alpha diversity analysis suggested that there was no significant difference on diversity within a sample between DM and CM (Simpson, p = 0.35; Shannon, p = 0.85). But the beta diversity analysis demonstrated that there was a robust difference on the gut microbiota compositions between DM and CM (Fig. 2A). Figure 2B showed the relative abundance of gut microbiota at the Family level. Using LEfSe analysis, 12 significantly increased OTUs and 59 significantly decreased OTUs responsible for discriminating DM from CM were identified (Fig. 2C). The data of these differential OTUs was described in Additional File 1. Interestingly, more than half of OTUs (43/71, 60.56%) belonged to phylum Firmicutes. Further analysis showed that these 43 OTUs mainly belonged to family Lachnospiraceae (20 OTUs), Oscillospiraceae (7 OTUs), Ruminococcaceae (6 OTUs) and Erysipelotrichaceae (3 OTUs). In addition, we found that 50 differential OTUs were significantly correlated with at least one DLB phenotype (Fig. 2C). Among these 50 differential OTUs, 28 OTUs belonged to phylum Firmicutes, and 12 of these 28 OTUs belonged to family Lachnospiraceae.

### Differential microbial genes in DM

**Fig. 3:**
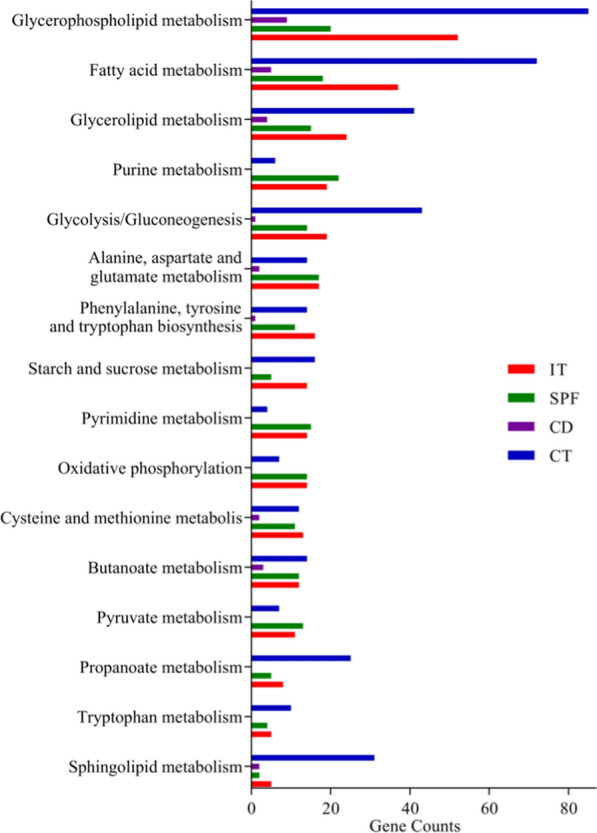
Differential metagenomic genes correlated with depressive-like behaviors. Depressive-like behaviors-related genes between depressed mice (n = 10) and control mice (n = 10) mainly clustered on three pathways: Glycerophospholipid metabolism, Fatty acids metabolism and Glycerolipid metabolism. *IT* immobility time, *CD* center distance, *SPF* sucrose preference, *CT* center time

The whole-genome shotgun sequencing of fecal samples was applied to identify the microbial genes. In total, 1534 increased genes and 1243 decreased genes in DM were identified. Correlation analysis showed that there were 280, 198, 29, and 401 differential genes significantly correlated with IT, SPF, CD, and CT, respectively. Functional analysis found that these DLB-related genes were mainly enriched in GP metabolism (Fig. 3). Meanwhile, we clustered the differential genes with a Pearson correlation coefficient > 0.9 into the co-abundant gene (CAG) groups; the CAG groups containing over 700 genes were identified as metagenomic species (MGS) [30]. Finally, 36 differential MGS (83.3% belonged to phylum Firmicutes) between the two groups were identified, and all of these MGS were significantly correlated with at least one DLB phenotype. These results demonstrated that the disordered phylum Firmicutes could be a hallmark of CUMS-induced depressed mice.

### Differential lipid metabolites in fecal and liver samples

**Fig. 4:**
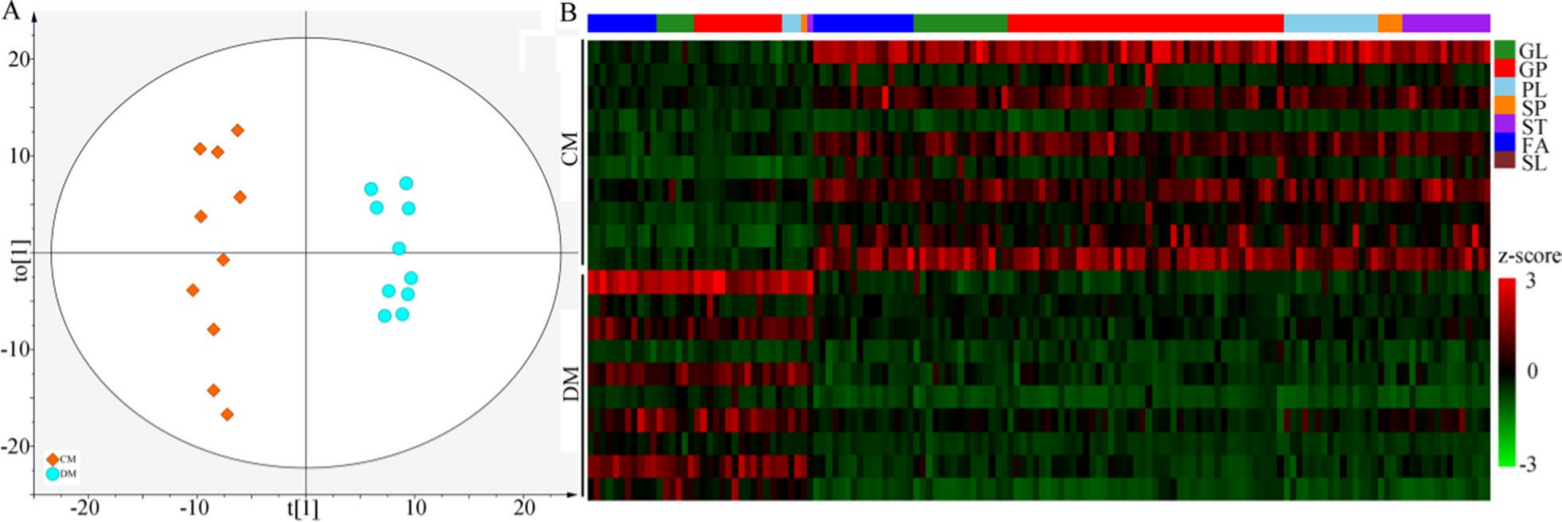
Differential fecal lipid metabolites between the two groups. **A** The scatter plot of OPLS-DA mode indicated that there were divergent metabolic phenotypes between DM (n = 10) and CM (n = 10) (see the explanations of t [1] and to [1] in Additional File 1); **B** The heat-map was built using the normalized abundance score for each differential metabolites. *DM* depressed mice, *CM* control mice, *GL* Glycerolipids, *GP* Glycerophospholipids, *PL* Prenol lipids, *SP* Sphingolipids, *ST* Steroids and steroid derivatives, *FA* Fatty acids, *SL* Saccharolipids

**Fig. 5:**
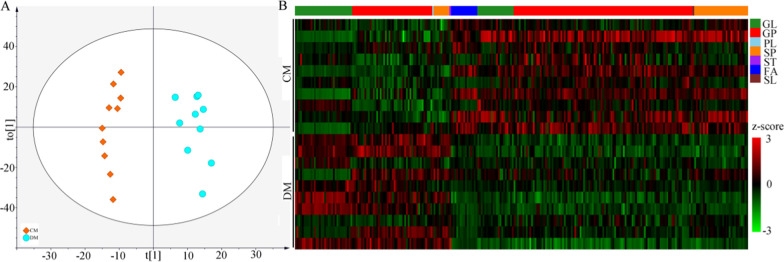
Differential liver lipid metabolites between the two groups. **A** The scatter plot of OPLS-DA mode indicated that there were divergent metabolic phenotypes between DM (n = 10) and CM (n = 10) (see the explanations of t [1] and to [1] in Additional File 1); **B** The heat-map was built using the normalized abundance score for each differential metabolites. *DM* depressed mice, *CM* control mice, *GL* Glycerolipids, *GP* Glycerophospholipids, *PL* Prenol lipids, *SP* Sphingolipids, *ST* Steroids and steroid derivatives, *FA* Fatty acids, *SL* Saccharolipids

Firstly, the lipid metabolites from the fecal samples were used to build the OPLS-DA model, and the built model suggested that the lipid metabolites could effectively separate DM from CM (Fig. 4A). By analyzing the corresponding OPLS-DA loading plot, 144 lipid metabolites with variable importance in projection (VIP) > 1.0 were viewed as the differential lipid metabolites responsible for separating DM from CM. These differential metabolites mainly belonged to GP (n = 58), fatty acids (FA) (n = 27) and glycerolipids (GL) (n = 21) (Additional File 1). Heat map of these lipid metabolites showed the consistent clustering pattern within the individual groups (Fig. 4B). Meanwhile, the pathway analysis showed that the GP metabolism in fecal sample was significantly affected. Secondly, the same analytical process was performed using the lipid metabolites from the liver samples. The results showed that: (i) the lipid metabolites in liver could also effectively separate DM from CM (Fig. 5A); (ii) the identified 255 lipid metabolites with VIP > 1.0 mainly belonged to GP (n = 146), GL (n = 52) and Sphingolipids (SP) (n = 39) (Fig. 5B) (Additional File 1); and (iii) the pathway analysis showed that GP metabolism in liver was significantly affected.

### Perturbed metabolic modules in DM

**Fig. 6:**
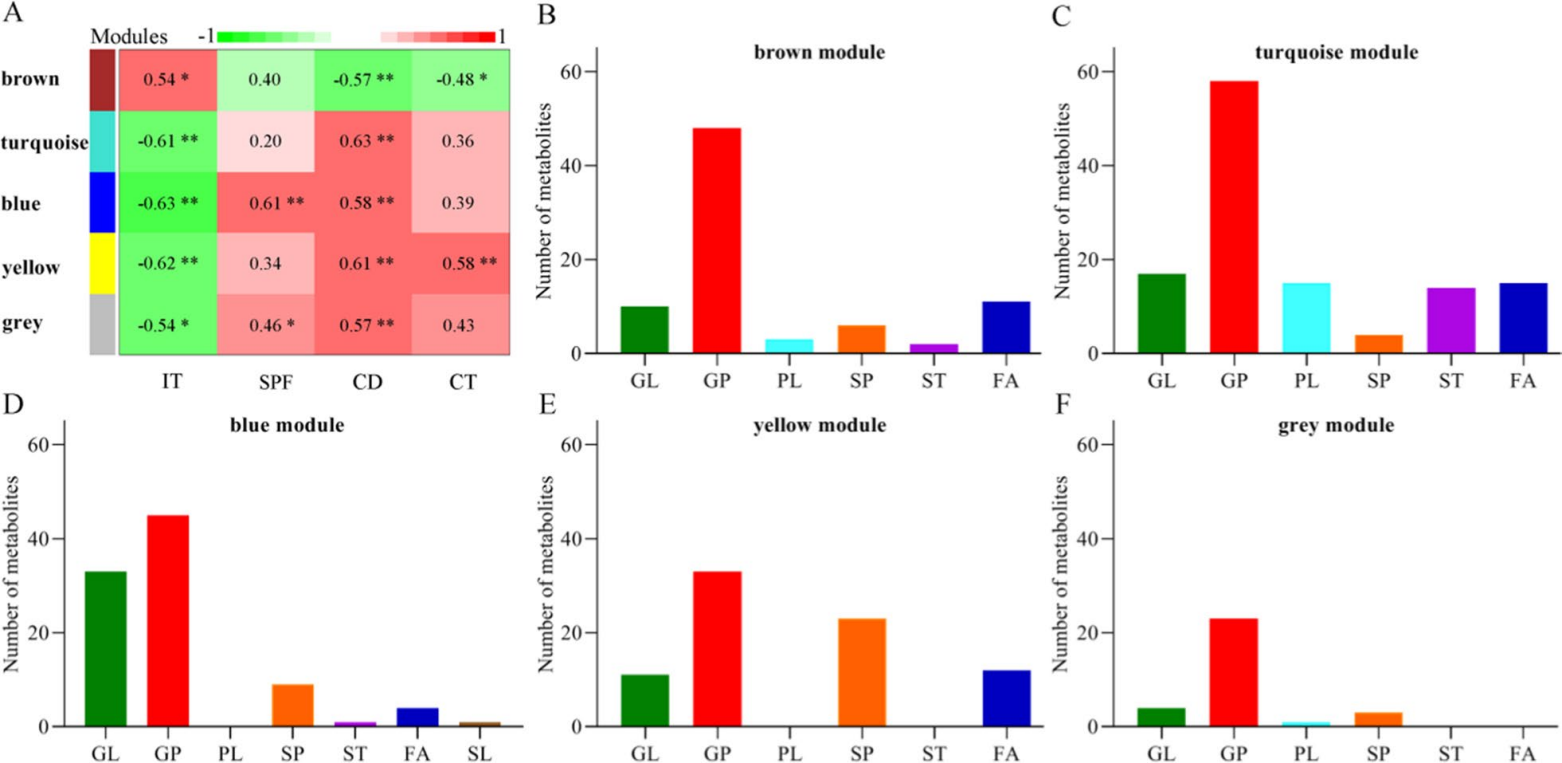
Metabolomic correlations with depressive-like behaviors in depressed mice. **A** The results of WGCNA showed that there were five modules significantly correlated with at least one DLB; **B**–**F** The histogram indicated the number of metabolites that was significantly correlated with DLB in each module. Data was from ten DM and ten CM. *IT* immobility time, *CD* center distance, *SPF* sucrose preference, *CT* center time, *GL* Glycerolipids, *GP* Glycerophospholipids, *PL* Prenol lipids, *SP* Sphingolipids, *ST* Steroids and steroid derivatives, *FA* Fatty acids, *SL* Saccharolipids

To find out the perturbed metabolic modules of Gut-Liver-Brain axis in DM, we used the WGCNA analysis to explore the relationships between DLB and these identified differential metabolites. In total, five different modules consisting of these differential lipid metabolites were identified (Fig. 6A). Each module was significantly correlated with at least one DLB phenotype. The differential metabolites from the brown module and turquoise module mainly belonged to GP metabolism (Fig. 6B, C). The differential metabolites from the blue module mainly belonged to GP and GL metabolism (Fig. 6D). The differential metabolites from the yellow module mainly belonged to GP and SP metabolism (Fig. 6E). The differential metabolites from the grey module mainly belonged to GP metabolism (Fig. 6F). These results suggested that the disturbance of GP metabolism had a close relationship with the onset of DLB.

### Differential neurotransmitters between DM and CM

**Fig. 7:**
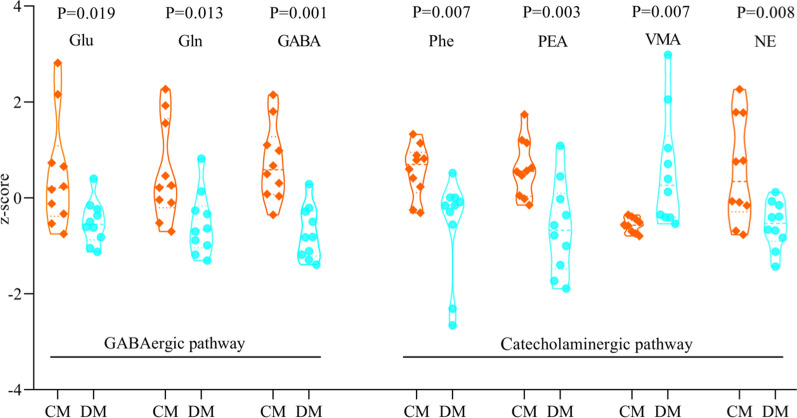
Seven significantly changed neurotransmitters in hippocampus. *DM* depressed mice, n = 10, *CM* control mice, n = 10, *Glu* glutamic acid, *Gln* glutamine, *GABA* gamma-aminobutyric acid, *Phe* l-phenylalanine, *PEA* phenylethylamine, *VMA* vanillylmandelic acid; *NE* norepinephrine

The following eight neurotransmitters in GABAergic pathway in the hippocampus were detected here: α-ketoglutaric acid, glutamic acid (Glu), ornithine, glutathione, glutamine (Gln), gamma-aminobutyric acid (GABA), succinic acid and aspartic acid. The following eight neurotransmitters in Catecholaminergic pathway in the hippocampus were detected here: l-phenylalanine (Phe), vanillylmandelic acid (VMA), 3,4-dihydroxyphenylacetic acid, norepinephrine (NE), phenylethylamine (PEA), L-3,4-dihydroxyphenylalanine, L-tyrosine, and tyramine. Finally, seven neurotransmitters were found to be significantly changed between DM and CM (Fig. 7). Compared to CM, DM was characterized by six decreased neurotransmitters (Glu, Gln, GABA, Phe, PEA and NE) and one increased neurotransmitter (VMA). These results indicated the disturbances of neurotransmitters in the hippocampus of DM.

### Comprehensive analysis of differential variables

**Fig. 8:**
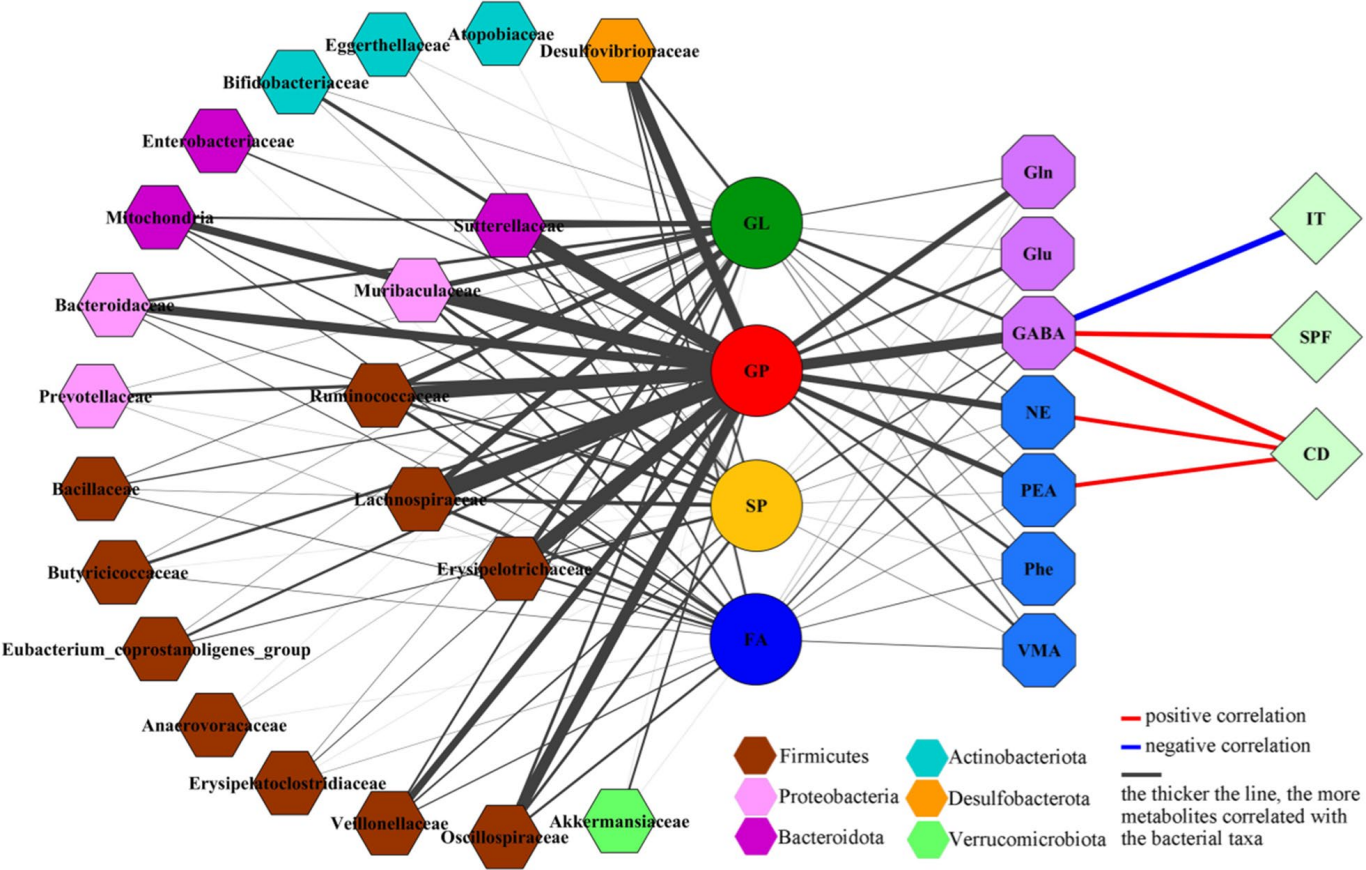
Correlations between differetial gut microbitoa, metabolites, neurotransmitters, and depressive-like behaviors. Data was from ten DM and ten CM. *IT* immobility time, *SPF* sucrose preference, *CD* center distance, *GL* Glycerolipids, *GP* Glycerophospholipids; *SP* Sphingolipids; *FA* Fatty acids; *Glu* glutamic acid; *Gln* glutamine; *GABA* gamma-aminobutyric acid; *Phe* l-phenylalanine; *PEA* phenylethylamine; *VMA* vanillylmandelic acid, *NE* norepinephrine

In total, there were 207 (51.11%), 75 (18.52%), 45 (11.11%), 42 (10.37%), and 36 (8.89%) metabolites belonged to GP, GL, SP, FA, and others, respectively. As shown in Fig. 8, the correlations between gut microbiota and lipid metabolites were mainly from phylum Firmicutes and GPs. There were significant relationships between most of GPs (n = 204) and bacterial taxa under phylum Firmicutes, especially family Lachnospiraceae (195 GPs), Ruminococcaceae (150 GPs), Erysipelotrichaceae (145 GPs) and Oscillospiraceae (121 GPs). Meanwhile, there were 165 GPs, 153 GPs and 124 GPs significantly correlated with family Muribaculaceae under phylum Bacteroidota, family Sutterellaceae under phylum Proteobacteria and family Desulfovibrionaceae under phylum Desulfobacterota, respectively.

**Fig. 9:**
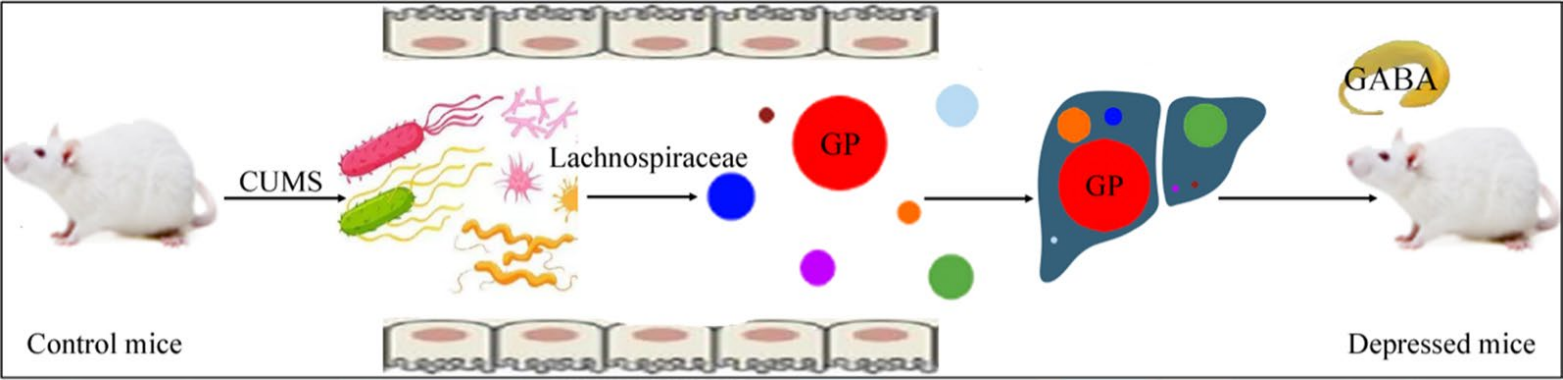
“Lachnospiraceae-GP metabolism-GABA” as potential way in Gut-Liver-Brain axis in CUMS-induced depressed mice. *CUMS* chronic unpredictable mild stress, *GP* Glycerophospholipids, *GABA* gamma-aminobutyric acid

The correlations between lipid metabolites and neurotransmitters were mainly from GPs and four neurotransmitters (Gln, GABA, PEA, and NE) (Fig. 8). The numbers of GPs significantly correlated with Glu, Gln, GABA, Phe, PEA, VMA and NE were 45, 71, 110, 33, 64, 32, 79, respectively. In addition, we found that GABA was significantly correlated with three DLB phenotypes (IT, r = − 0.69; SP, r = 0.52, and CD, r = 0.56); the CD was also significantly correlated with PEA (r = 0.46) and NE (r = 0.46) (Fig. 8). These results showed that the disturbance of Firmicutes, especially family Lachnospiraceae, might participate in the onset of DLB by affecting hippocampal neurotransmitters, especially GABA, via GP metabolism (Fig. 9). Based on these results, we suggested that Lachnospiraceae-GP metabolism-GABA might be a potential way between gut and brain in CUMS-induced depressed mice.

## Discussion

The relationship between the onset of depression and gut microbiota has been investigated for decades, but the specific pathways to link the gut and brain are still unclear. In this study, we observed that the DM was characterized by alterations of gut microbial compositions and functions, metabolic pathways in fecal and liver samples, and hippocampal neurotransmitters. The altered microbial and metabolic modules mainly linked the Firmicutes with the disordered of GP metabolism in DM: family Lachnospiraceae was significantly correlated with 94.20% differential GPs. Meanwhile, the seven differential neurotransmitters were mainly significantly correlated with GPs, and GABA was significantly correlated with three main DLB phenotypes. Taken together, we deduced that the abnormal Firmicutes might mediate DLB in DM by affecting host’s GP metabolism, and “Lachnospiraceae-GP metabolism-GABA” might be the potential way for gut microbiota participating in the onset of depression.

In our previous study, we found that the disordered gut microbiota might played an important role in causing DLB via GP metabolism [29]. Compared to our previous findings, the results in the present study had several advantages. Firstly, liver had a vital role in the crosstalk of gut and brain. The metabolic phenotypes in liver were explored here, but not in our previous study [29]. Secondly, we further found here that Lachnospiraceae under Firmicutes might be the main differential bacterial taxa that affected GP in depression. Thirdly, in our previous study, we could not identify the specific neurotransmitters in tryptophan pathway; here, we found that GABA in GABAergic pathway was mainly affected by GP metabolism. Thus, our current findings were the great supplement and sublimation to our previous findings [29].

Firmicutes is one of the two major phyla making up the gut microbiota in humans, and the other one is Bacteroidetes [32]. Many researches reported that these two bacterial phyla might be the hallmark of depression [33, 34]. In our previous study, we found that there were more than half (82.34%) of differential genera in depression patients compared to healthy controls belonging to phylum Firmicutes [11]. Consistent with clinical findings, using CRS-induced depression mouse model, we observed that the differences on gut microbiota between DM and CM could be largely explained by the disturbance of phylum Firmicutes [13, 29]. Using the depressed *Macaca fascicularis* model, our group found that the disordered phylum Firmicutes could be a hallmark of depressed monkeys [30]. Here, 71 differential OTUs were identified, and 60.56% differential OTUs belonged to the phylum Firmicutes. Thus, our current findings supported the opinion that the dysbiosis of Firmicutes was a distinguishing feature of depression.

As the important components of neural membrane, GPs have many essential functions, such as signal transmission and modulation of enzyme activity. The changes of GPs in neural membrane have been found in neurological disorders [35]. Yu et al. reported that GPs could play a key role in inducing depressive- and anxiety-like behaviors [36]. Liu et al. found the disturbance of GP metabolism in the plasma of depression patients, and identified several GPs as potential biomarkers to diagnose depression [37]. In our previous studies, we found the changes of some GPs in the urine and serum of depression patients [18–20] and in the liver and colon of depressed mice [22, 28]. Researchers have reported that there are obvious disturbances of lipid metabolism in depression [38, 39], but its specific role in the onset of depression is still unclear. Here, 51.1% differential lipid metabolites belonged to GP, and the main metabolic pathway that both microbial and metabolic modules were involved in was GP metabolism. These results suggested that GP metabolism might be the bridge between gut microbiota and depression.

As the largest solid organ for energy metabolism, liver has a vital role in many biological processes, such as oxidative stress and inflammation [38–40]. Although there are obvious disturbances of lipid metabolism in depression [41, 42] and liver has a vital role in lipid metabolism, few studies have explored the role of liver metabolism on depression. Due to the unique vascular system of liver, it is vulnerable to exposure to the metabolic products of gut microbiota [43]. In our previous study, we found that “depression microbes” from depression patients could impact on liver metabolism of germ-free mice [21]. Le Strat et al. observed the close association of liver disease with both suicide attempts and depression [44]. Here, we found that the liver lipid metabolism, especially GP metabolism, was significantly correlated to gut microbiota. Our findings indicated that liver was an important node in the crosstalk between gut and brain in depression.

Nowadays, the underlying molecular mechanisms between depression and gut microbiota are not clearly demonstrated yet. Some researchers reported that the imbalance of gut microbiota might participate in the onset of depression by affecting the level of neurotransmitters, such as serotonin [13, 45, 46]. Other researches linked the disturbance of gut microbiota to the decreased level of GABA, which was known to be implicated in depression [47]. Our previous work suggested that the disordered Firmicutes might play a role in the onset of depression through regulating the inflammatory response [11]. Interestingly, some GPs were viewed as the modulator of inflammatory response [21, 48]. Using germ-free mice, we found that the disturbance of gut microbiota might induce DLB through regulating host’s metabolism [14]; further research showed that the gut microbiota mainly affected the host’s GP metabolism [29]. In addition, although no correlation between Lachnospiraceae and GP metabolism was identified, Zhang et al. reported that the differential metabolites in the feces of type 2 diabetes mellitus rats were mainly involved in the pathway of GP metabolism, and some members of Lachnospiraceae were significantly changed [49]. Zheng et al. found that the differential brain metabolites in a nonhuman primate model of depression were mainly involved in hippocampal GP metabolism, and there were five differential OTUs belonged to Lachnospiraceae [30]. In the present study, most of differential GPs were found to be significantly correlated with Lachnospiraceae and GABA. Taken together, we suggested that “Lachnospiraceae-GP metabolism-GABA” might be a potential way in Gut-Liver-Brain axis in DM.

Several limitations should be mentioned here. Firstly, we only assessed the relationships between gut microbiota and neurotransmitters in Gut-Liver-Brain axis. Considering the effects of gut microbiota on inflammatory response, future studies should be conducted to explore how the disturbances of Firmicutes affected the inflammatory response in hippocampus via GP metabolism. Secondly, in our previous study, we found that there might be age- and sex-specific differential changes on gut microbiota compositions in depression patients [12]. In this study, only adult male mice were used, thus the age-and sex-bias could not be ruled out. Thirdly, only the hippocampal area of the brain was studied here; other brain regions, such as prefrontal cortex, could also mediate interest, stress and mood. Therefore, other brain regions were also worthy of studying to find out more novel insights on the crosstalk between gut microbiota and brain. Fourthly, we did not consider the off target effects of Lachnospiraceae in this study; future studies should explore whether or not Lachnospiraceae could be viewed as a treatment target for depression. Fifthly, due to the limitations of technologies and funds, we did not conduct vitro experiments in cell lines to validate our findings and delineate relevant molecular mechanisms. Future studies should be performed to support our results and explore the relevant molecular mechanisms in Gut-Liver-Brain axis in depression. Sixthly, the current findings were restricted to samples from mice; thus future studies should be conducted to validate our findings in patient samples, at least some key findings. Seventhly, we did not use mutant mice model to validate some of our findings. Future studies could use mutant mice model to decrease the level of GABA, and observe whether it could affect behaviors of mice.

## Conclusions

In conclusion, using CUMS-induced mice depression model, we observed that the depressed mice were characterized by the disturbance of gut microbiota compositions, microbial functions, GP metabolism in liver, and GABAergic and Catecholaminergic pathways in hippocampus. Meanwhile, we found a potential way “Lachnospiraceae-GP metabolism-GABA” in Gut-Liver-Brain axis in depressed mice. The results could further improve our understanding of the specific mechanism of action of gut microbiota in the onset of depression.

## Supplementary Information

**Additional file 1: Table S1.** CUMS stressors in every week of CUMS procedure. **Table S2.** Results of behavioral tests in the two groups. **Table S3.** Differential OTUs between the two groups. **Table S4.** Differential fecal metabolites between the two groups. **Table S5.** Differential liver metabolites between the two groups. **Table S6.** Differential neurotransmitters between the two groups.

## Data Availability

The data can be accessed from corresponding author or first author upon request.

## Abbreviations

CUMS: Chronic unpredictable mild stress
DM: Depressed mice
CM: Control mice
DLB: Depressive-like behaviors
WGCNA: Weighted correlation network analysis
OTU: Operational taxonomic unit
CNS: Central nervous system
BW: Body weight
SPF: Sucrose preference
OFT: Open field test
FST: Forced swim test
SPT: Sucrose preference test
CD: Center area
CT: Center area
IT: Immobility time
LC-MS: Liquid chromatography-mass spectrometry
PCoA: principal coordinate analysis
LEfSe: Linear discriminant analysis effective size
OPLS-DA: Orthogonal partial least squares discriminant analysis
CAG: Co-abundant gene
MGS: Metagenomic species
VIP: Variable importance in projection
Glu: Glutamic acid
Gln: Glutamine
GABA: Gamma-aminobutyric acid
Phe: l-phenylalanine
PEA: Phenylethylamine
VMA: Vanillylmandelic acid
NE: Norepinephrine
GL: Glycerolipids
GP: Glycerophospholipids
PL: Prenol lipids
SP: Sphingolipids
ST: Steroids and steroid derivatives
FA: Fatty acids
SL: Saccharolipids
NIH: National Institutes of Health
