# Supplementary material for: Multi-omics data reveals the important role of glycerophospholipid metabolism in the crosstalk between gut and brain in depression
Source: J Transl Med. 2023 Feb 7;21:93. doi: 10.1186/s12967-023-03942-w (PMC9903503; doi:10.1186/s12967-023-03942-w)
Supplement: Supplementary file 1 — Additional file 1: Table S1. CUMS stressors in every week of CUMS procedure. Table S2. Results of behavioral tests in the two groups. Table S3. Differential OTUs between the two groups. Table S4. Differential fecal metabolites between the two groups. Table S5. Differential liver metabolites between the two groups. Table S6. Differential neurotransmitters between the two groups. [file 12967_2023_3942_MOESM1_ESM.docx]

**Additional file Methods**

***1. CUMS procedure***

In this study, the chronic unpredictable mild stress (CUMS) procedure here was mainly performed in accordance with the procedures in our previous studies [1, 2]. Briefly, the mice in the experiment group were received CUMS for four weeks. Each mouse received one or two different stressors per day, and the same stressor was not allowed to use for two consecutive days. At last, the food and water were deprived for 24 hours before conducting behavioral experiments. The mice in the control group were not disturbed and could freely obtain food and water. The CUMS stressors in every week of CUMS procedure was as following:

**Table S1 CUMS stressors in every week of CUMS procedure**

|  | Sunday | Monday | Tuesday | Wednesday | Thursday | Friday | Saturday |
| --- | --- | --- | --- | --- | --- | --- | --- |
| Week1 | CT-24 | SW | LOSL | LOSW | LOTN | WC | FWD |
| Week2 | LOSL | LOTN | 30-IF | SW | FWD | LOSW | CT-24 |
| Week3 | R-4 | FWD | WC | LOTN | SW | LOSL | LOSW |
| Week4 | SW | CT-24 | LOTN | LOSW | R-4 | 30-IF | FWD |

Abbreviations: CT-24, cages tilted 45°for 24h; SW, swimming in 4℃ water for 5 minutes, wet bedding for 24h; LOSL, lights on overnight and strobe light on for 12h; LOSW, lights on overnight and swimming in 45℃ water for 5 minutes; LOTN, lights on overnight and tail nipped for 1 minute or shaking for 10 minutes; WC, wet cages; FWD, food and water deprivation for 24h; 30-IF, 30 inescapable footshocks; R-4, restraint for 4h.

***2. Behaviors testing methods***

The procedures of behaviors experiments were exactly performed in accordance with the procedures in our previous studies [1-3]. Briefly, in open field test (OFT), we put the mice into the center of box (45 x 45 x 45 cm) to freely explore the box for six minutes. The data produced in last five minutes were collected, including total distance, center time and center distance. In forced swim test (FST), we put the mice into a plexigas cylinder (15 cm diameter x 30 cm height) for six minutes. The plexiglas cylinder was 15 cm diameter and 30 cm height and filled with 18 cm height of water. The temperature of water was 24 ±1°C. The immobility time in last five minutes was collected. In sucrose preference test (SPT), firstly, the mice were trained to adapt to 1% sucrose solution before SPT; secondly, after adaption, the mice were freely access to 1% sucrose solution or water. The consumptions of 1% sucrose solution and water were collected. The sucrose preference was defined as the proportion of 1% sucrose solution consumption in the total liquid consumptions.

***3. 16S rRNA gene sequence and Metagenomic analysis***

The procedures of 16S rRNA gene sequence analysis were exactly performed in accordance with the procedures in our previous studies [3-6]. Briefly, after obtaining the raw gene sequences, the Mothur (Version 1.31.2, <http://www.mothur.org/>) was used here to collect unique reads: raw gene sequences with any barcode mismatches, homopolymer runs exceeding six bases, primer mismatches, ambiguous bases and <200 bp or >1000 bp were excluded. Then, we assigned the remained sequences (>=97% pair-wise sequence identity) into operational taxonomic units (OTUs). At last, we used Ribosomal Database Project (RDP) reference database to taxonomically classify the OTUs. Meanwhile, the procedures of metagenomic analysis were exactly performed in accordance with the procedures in our previous studies [3-6]. Briefly, Bayesing model averaging was used here to remove the reads that belonged to the reference genome of mouse. Then, we aligned the metagenomic genes into the Kyoto Encyclopedia of Genes and Genomes (KEGG) genes using BLASTP (expectation value, 1e-5). Finally, we used the KEGG to annotate the function of the identified nonredundant genes.

***4. Fecal sample preparation***

The procedures of fecal sample preparation were exactly performed in accordance with the procedures in our previous studies [3-5]. Firstly, we weighed and homogenized fecal sample (50 mg) in 500 μl methanol, which contained known amount of internal standard. Secondly, the mixture was centrifugated (4°C, 15 minutes, and 14,000 rpm), and the obtained supernatant was added into a new tube and dried under vacuum under 30 °C. Thirdly, after adding 20 μl acetonitrile into the obtained residue, the mixture was vortexed for 30 seconds and then vortexed again with 80 μl water for 30 seconds. Finally, the mixture was centrifugated (4°C, 15 minutes, and 14,000 rpm), and then we added 80 μl supernatant into a glass vial for later LC-MS analysis.

***5. Liver sample preparation***

Briefly, the procedure of liver sample preparation was as following: Firstly, using methanol-water (1/1, v/v) (800 μl) to transfer liver sample (30 mg) into 1.5ml centrifuge tube to conduct homogenate. Secondly, transferred homogenized mixture (800 μl) and chloroform (800 μl) into a glass centrifuge tube and vortexed for 30 seconds. Thirdly, after ultrasonic extraction for 600 seconds and then standing for 900 seconds at 4°C, the mixture was centrifugated (4°C, 900 seconds, and 2,000 rpm). Fourthly, collected the lower chloroform layer after liquid layering and added it into a high recovery glass tube for vacuum volatilization. Fifthly, isopropanol-methanol (2/1, v/v) (1 ml) was added into the original centrifuge tube, then vortexed for 30 seconds and conducted ultrasonic extraction for 600 seconds. Sixthly, after standing for 900 seconds at 4°C and then centrifugating (4°C, 900 seconds, and 2,000 rpm), collected the lower chloroform layer after liquid layering and added it into a high recovery glass tube for vacuum volatilization. Seventhly, mingled the obtained lipid residue and isopropanol-methanol (1:1, v/v) (300 ul). Eighthly, after conducting vortex for 30 seconds and ultrasonic extraction for 180 seconds, added the mixture into 1 ml centrifuge tube for centrifugation (4°C, 600 seconds, and 12,000 rpm). Finally, added the obtained supernatant (200μl) into a injection vial for later LC-MS analysis.

***6. LC-MS analysis***

The procedures of LC-MS analysis were exactly performed in accordance with the procedures in our previous studies [3-5]. The metabolomic analysis of the sample extracts was conducted on an ACQUITY I Class UPLC system coupled to a Waters G2-S QTOF system (Waters, Milford, MA, USA), with a Waters ACQUITY UPLC HSS T3 column (2.1 × 100 mm, 1.8 μm) and a Waters ACQUITY UPLC HSS T3 VanGuard precolumn (2.1×5 mm, 1.8 μm). 5 μL of extract was injected into the system for metabolomic fingerprinting. A 25.5-min gradient at 450 μL/min was adopted. Formic acid (0.1%) and ammonium formate (5 mmol/L, pH 9) were utilized as mobile phase A in the positive and negative modes, respectively. Acetonitrile was used as mobile phase B in both modes. Following gradient sets were applied: 0-1 min, 2-5% B; 1-3 min, 5-40% B; 3-17 min, 40-98% B; 17-23 min, 98% B; 23-23.1 min, 98-2% B, 23.1-25.5 min, 2% B. The G2-S QTOF system (Waters, Milford, MA, USA) was set in MSE mode at a resolution of 30,000 and a scan rate of 0.2 sec in the mass range from 50 to 2000, with running parameters for both of the positive and negative modes as described below: 3 kV capillary voltage, 40 V cone voltage, 80 V source offset, 120 °C source temperature, 40 °C desolvation temperature, 5 h/L cone gas flow and 800 L/h desolvation gas flow. The identification of different metabolites in our samples was conducted through Progenesis QI (Waters, Milford, MA, USA), a software which enables the performance of peak alignment, peak selection, deconvolution as well as the identification of metabolites against library databases including HMDB, ChemSpider, LipidBlast, METLIN, and CCS etc.

***7. Neurotransmitters detection***

The procedures of neurotransmitters detection in hippocampus samples were exactly performed in accordance with the procedures in our previous studies [3, 4]. Firstly, the hippocampus samples preparation was conducted: i) i) accurately weighed the sample, then transferred the sample into a 1.5 ml Eppendorf tube; ii) added 400 μl mixture of methanol-water (4/1, v/v), the 2-chloro-l-phenylalanine (75 ng/mL) dissolved in methanol was viewed as internal standard; iii) conducted homogenate under low temperature for 10 minutes, then conducted ultrasonic extraction in ice water bath for 10 minutes; iv) after centrifugation (10 minutes, 14000 rpm, 4°C), transferred 300 ul supernatant into a new 1.5 ml Eppendorf tube; v) added 200 μl mixture of methanol-water (4/1, v/v) into the original Eppendorf tube, then conduct vortex for 30 seconds and ultrasonic extraction in ice water bath for 5 minutes; vi) after centrifugation (10 minutes, 14000 rpm, 4°C), transferred 200 ul supernatant into the new 1.5 ml Eppendorf tube; vii) after drying in a freeze concentration centrifugal dryer, added methanol-water(4/1, v/v) into the resultant powder, and then conducted vortex for 30 seconds and ultrasonic extraction for 3 minutes; viii) after centrifugation (10 minutes, 14000 rpm, 4°C), transferred 150μl supernatant into the injection vial for later LC-MS analysis. The quantification of targeted neurotransmitters was performed on a Waters Acquity UPLC system.

**Additional file Results**

***1. Results of behavioral tests***

**Table S2 Results of behavioral tests in the two groups**

|  | TD | CD (%) | CT (%) | BW-0 | BW-4 | IT | SPF-0 | SPF-4 |
| --- | --- | --- | --- | --- | --- | --- | --- | --- |
| CM | 1367.89(334.27) | 11.53(3.45) | 6.71(3.82) | 22.24(1.09) | 25.24(1.04) | 28.47(21.20) | 0.85(0.04) | 0.87(0.05) |
| DM | 1241.61(216.83) | 4.72(4.33) | 2.27(3.36) | 22.19(1.30) | 24.11(1.78) | 88.69(32.32) | 0.86(0.06) | 0.80(0.04) |
| p | 0.3300 | 0.0011 | 0.0129 | 0.9385 | 0.1010 | 0.0001 | 0.6409 | 0.0079 |

Abbreviations: CM, control mice; DM, depressed mice; TD, total distance (cm); CD(%), center distance(%);CT(%), center time (%);BW-0, body weight at baseline; BW-4, body weight at week 4; IT, immobility time; SPF-0, sucrose preference at baseline; SPF-4, sucrose preference at week 4.

***2. Differential OTUs***

**Table S3 Differential OTUs between the two groups**

| **OTU ID** | **Phylum** | **Family** | **p** | **LDA** | **CM** |  | **DM** |  |
| --- | --- | --- | --- | --- | --- | --- | --- | --- |
|  |  |  |  |  | **Mean(%)** | **SD** | **Mean(%)** | **SD** |
| OTU103 | Firmicutes | Ruminococcaceae | 0.0002 | 5.68 | 0.0154 | 0.0102 | 0.0846 | 0.0846 |
| OTU751 | Firmicutes | Bacillaceae | 0.0413 | 5.49 | 0.0413 | 0.0397 | 0.0587 | 0.0587 |
| OTU715 | Firmicutes | Lactobacillaceae | 0.011 | 5.4 | 0.0221 | 0.033 | 0.0779 | 0.0779 |
| OTU637 | Actinobacteriota | Atopobiaceae | 0.0052 | 5.25 | 0.0178 | 0.0144 | 0.0822 | 0.0822 |
| OTU88 | Actinobacteriota | Eggerthellaceae | 0.0423 | 4.68 | 0.0071 | 0.0104 | 0.0929 | 0.0929 |
| OTU642 | Firmicutes | Ruminococcaceae | 0.0164 | 4.43 | 0.0233 | 0.059 | 0.0767 | 0.0767 |
| OTU587 | Bacteroidota | Muribaculaceae | 0.0494 | 4.19 | 0.0331 | 0.027 | 0.0669 | 0.0669 |
| OTU43 | Desulfobacterota | Desulfovibrionaceae | 0.0215 | 4.08 | 0.0274 | 0.0614 | 0.0726 | 0.0726 |
| OTU278 | Firmicutes | Anaerovoracaceae | 0.0423 | 3.59 | 0.038 | 0.0712 | 0.062 | 0.062 |
| OTU743 | Firmicutes | Erysipelatoclostridiaceae | 0.0423 | 3.46 | 0.0225 | 0.0342 | 0.0775 | 0.0775 |
| OTU534 | Firmicutes | Lachnospiraceae | 0.013 | 3.37 | 0 | 0 | 0.1 | 0.1 |
| OTU194 | Firmicutes | Erysipelotrichaceae | 0.0306 | 2.97 | 0 | 0 | 0.1 | 0.1 |
| OTU73 | Firmicutes | Lachnospiraceae | 0.013 | -2.94 | 0.1 | 0.1237 | 0 | 0 |
| OTU575 | Desulfobacterota | Desulfovibrionaceae | 0.013 | -2.94 | 0.1 | 0.1237 | 0 | 0 |
| OTU85 | Firmicutes | norank_o__Clostridia_vadinBB60_group | 0.0306 | -3.02 | 0.1 | 0.1424 | 0 | 0 |
| OTU313 | Firmicutes | Lachnospiraceae | 0.0333 | -3.02 | 0.0854 | 0.0951 | 0.0146 | 0.0146 |
| OTU122 | Firmicutes | Oscillospiraceae | 0.013 | -3.08 | 0.1 | 0.1205 | 0 | 0 |
| OTU371 | Firmicutes | Lachnospiraceae | 0.013 | -3.08 | 0.1 | 0.1142 | 0 | 0 |
| OTU659 | Firmicutes | Ruminococcaceae | 0.0052 | -3.09 | 0.1 | 0.1111 | 0 | 0 |
| OTU224 | Firmicutes | Oscillospiraceae | 0.047 | -3.11 | 0.0763 | 0.0595 | 0.0237 | 0.0237 |
| OTU557 | Firmicutes | Lachnospiraceae | 0.0386 | -3.13 | 0.0912 | 0.1081 | 0.0088 | 0.0088 |
| OTU509 | Firmicutes | Ruminococcaceae | 0.0238 | -3.14 | 0.0819 | 0.0692 | 0.0181 | 0.0181 |
| OTU563 | Firmicutes | Oscillospiraceae | 0.015 | -3.16 | 0.0897 | 0.0818 | 0.0103 | 0.0103 |
| OTU290 | Bacteroidota | Bacteroidaceae | 0.013 | -3.21 | 0.1 | 0.1122 | 0 | 0 |
| OTU643 | Firmicutes | Erysipelotrichaceae | 0.0192 | -3.21 | 0.076 | 0.0536 | 0.024 | 0.024 |
| OTU451 | Firmicutes | Eubacterium_coprostanoligenes_group | 0.0103 | -3.22 | 0.0763 | 0.0561 | 0.0237 | 0.0237 |
| OTU744 | Firmicutes | Lachnospiraceae | 0.0118 | -3.24 | 0.0919 | 0.11 | 0.0081 | 0.0081 |
| OTU151 | Firmicutes | Lachnospiraceae | 0.0211 | -3.27 | 0.0896 | 0.1023 | 0.0104 | 0.0104 |
| OTU596 | Firmicutes | Lachnospiraceae | 0.0333 | -3.27 | 0.086 | 0.0953 | 0.014 | 0.014 |
| OTU374 | Firmicutes | Oscillospiraceae | 0.0306 | -3.31 | 0.1 | 0.1401 | 0 | 0 |
| OTU782 | Firmicutes | Lachnospiraceae | 0.0019 | -3.32 | 0.1 | 0.1 | 0 | 0 |
| OTU410 | Proteobacteria | Sutterellaceae | 0.0306 | -3.32 | 0.1 | 0.1538 | 0 | 0 |
| OTU551 | Proteobacteria | Mitochondria | 0.0333 | -3.33 | 0.0938 | 0.1852 | 0.0062 | 0.0062 |
| OTU826 | Firmicutes | Oscillospiraceae | 0.013 | -3.36 | 0.1 | 0.1139 | 0 | 0 |
| OTU807 | Bacteroidota | Prevotellaceae | 0.013 | -3.36 | 0.1 | 0.1261 | 0 | 0 |
| OTU35 | Firmicutes | Oscillospiraceae | 0.0131 | -3.38 | 0.0878 | 0.0791 | 0.0122 | 0.0122 |
| OTU689 | Firmicutes | Lachnospiraceae | 0.013 | -3.4 | 0.0957 | 0.0903 | 0.0043 | 0.0043 |
| OTU220 | Firmicutes | Lachnospiraceae | 0.013 | -3.44 | 0.1 | 0.1375 | 0 | 0 |
| OTU163 | Proteobacteria | Enterobacteriaceae | 0.0451 | -3.44 | 0.0954 | 0.1453 | 0.0046 | 0.0046 |
| OTU485 | Firmicutes | Ruminococcaceae | 0.0406 | -3.46 | 0.089 | 0.0908 | 0.011 | 0.011 |
| OTU717 | Firmicutes | Lachnospiraceae | 0.013 | -3.49 | 0.1 | 0.1675 | 0 | 0 |
| OTU652 | Firmicutes | Lachnospiraceae | 0.0189 | -3.51 | 0.0757 | 0.054 | 0.0243 | 0.0243 |
| OTU721 | Firmicutes | Ruminococcaceae | 0.0135 | -3.55 | 0.0786 | 0.051 | 0.0214 | 0.0214 |
| OTU467 | Firmicutes | Lachnospiraceae | 0.0255 | -3.57 | 0.0888 | 0.1042 | 0.0112 | 0.0112 |
| OTU204 | Firmicutes | Oscillospiraceae | 0.0211 | -3.59 | 0.0906 | 0.1121 | 0.0094 | 0.0094 |
| OTU649 | Proteobacteria | Sutterellaceae | 0.0093 | -3.62 | 0.0923 | 0.1103 | 0.0077 | 0.0077 |
| OTU498 | Firmicutes | Lachnospiraceae | 0.0306 | -3.66 | 0.1 | 0.2493 | 0 | 0 |
| OTU635 | Firmicutes | Lachnospiraceae | 0.003 | -3.67 | 0.0836 | 0.0502 | 0.0164 | 0.0164 |
| OTU241 | Bacteroidota | Muribaculaceae | 0.036 | -3.67 | 0.0971 | 0.1751 | 0.0029 | 0.0029 |
| OTU327 | Proteobacteria | Sutterellaceae | 0.0081 | -3.72 | 0.0954 | 0.1555 | 0.0046 | 0.0046 |
| OTU24 | Firmicutes | Butyricicoccaceae | 0.0071 | -3.79 | 0.0884 | 0.0995 | 0.0116 | 0.0116 |
| OTU237 | Actinobacteriota | Eggerthellaceae | 0.0176 | -3.85 | 0.08 | 0.1276 | 0.02 | 0.02 |
| OTU730 | Desulfobacterota | Desulfovibrionaceae | 0.0215 | -3.86 | 0.085 | 0.0815 | 0.015 | 0.015 |
| OTU621 | Firmicutes | Veillonellaceae | 0.0052 | -3.87 | 0.1 | 0.2513 | 0 | 0 |
| OTU442 | Firmicutes | Lachnospiraceae | 0.0043 | -3.95 | 0.0986 | 0.1236 | 0.0014 | 0.0014 |
| OTU729 | Desulfobacterota | Desulfovibrionaceae | 0.0323 | -3.95 | 0.0854 | 0.1004 | 0.0146 | 0.0146 |
| OTU11 | Proteobacteria | Sutterellaceae | 0.0034 | -3.98 | 0.0884 | 0.0656 | 0.0116 | 0.0116 |
| OTU824 | Bacteroidota | Muribaculaceae | 0.0255 | -3.98 | 0.0872 | 0.0906 | 0.0128 | 0.0128 |
| OTU366 | Bacteroidota | Muribaculaceae | 0.036 | -4.03 | 0.0983 | 0.1463 | 0.0017 | 0.0017 |
| OTU780 | Bacteroidota | Muribaculaceae | 0.0038 | -4.1 | 0.0938 | 0.1098 | 0.0062 | 0.0062 |
| OTU446 | Bacteroidota | Muribaculaceae | 0.0044 | -4.1 | 0.0854 | 0.0639 | 0.0146 | 0.0146 |
| OTU296 | Firmicutes | Lachnospiraceae | 0.036 | -4.16 | 0.0992 | 0.2406 | 0.0008 | 0.0008 |
| OTU505 | Firmicutes | Erysipelotrichaceae | 0.0123 | -4.22 | 0.0924 | 0.1196 | 0.0076 | 0.0076 |
| OTU795 | Bacteroidota | Muribaculaceae | 0.0182 | -4.27 | 0.0831 | 0.0776 | 0.0169 | 0.0169 |
| OTU234 | Firmicutes | Lachnospiraceae | 0.013 | -4.29 | 0.1 | 0.2388 | 0 | 0 |
| OTU81 | Actinobacteriota | Bifidobacteriaceae | 0.0191 | -4.34 | 0.0902 | 0.1221 | 0.0098 | 0.0098 |
| OTU160 | Bacteroidota | Muribaculaceae | 0.0205 | -4.4 | 0.0979 | 0.1442 | 0.0021 | 0.0021 |
| OTU14 | Bacteroidota | Muribaculaceae | 0.0451 | -4.49 | 0.0915 | 0.1379 | 0.0085 | 0.0085 |
| OTU772 | Bacteroidota | Muribaculaceae | 0.0412 | -4.92 | 0.0809 | 0.0651 | 0.0191 | 0.0191 |
| OTU539 | Bacteroidota | Muribaculaceae | 0.0412 | -5.08 | 0.0804 | 0.0622 | 0.0196 | 0.0196 |
| OTU437 | Verrucomicrobiota | Akkermansiaceae | 0.0478 | -5.39 | 0.0997 | 0.1971 | 0.0003 | 0.0003 |

***3.*** ***Differential metabolites***

The OPLS-DA model was used to generate the plots shown in figure 4A and 5A. The OPLS-DA model of the two-class separation (discrimination) problem will always have one predictive component and no, one or more orthogonal components. In the case of a 1 + 1 model, or any higher number of orthogonal components, the default score scatter plot in SIMCA is t[1] (x-axis) vs. to[1] (y-axis). The scatter plot of t[1] vs. to[1] is a window in the X space in which the separation of the two classes of observations occurs in the horizontal (t[1]) direction. Hence, it is sufficient to interpret the corresponding loading, p1, to uncover the variables with class discriminating ability. The vertical (to[1]) direction expresses within class variability, which is unrelated to the question (i.e., to discriminate between the two classes), but which is important for the total understanding of the problem. In this study, the built OPLS-DA mode yielded 144 differential fecal metabolites (Supplementary Table 4) and 261 differential liver metabolites (Supplementary Table 5).

**Table S4 Differential fecal metabolites between the two groups**

| Metabolites | CM |  | DM |  | p |
| --- | --- | --- | --- | --- | --- |
|  | Mean | SD | Mean | SD |  |
| PE(42:9) | 0.3524 | 0.2368 | 3.3927 | 3.8183 | 0.0217 |
| LysoPC(16:0) | 0.0198 | 0.011 | 0.1241 | 0.1071 | 0.0067 |
| Trilobinol | 38.4168 | 47.4646 | 229.81 | 219.718 | 0.0149 |
| LysoPS(18:0/0:0) | 0.0378 | 0.0461 | 0.2231 | 0.1596 | 0.0024 |
| TAG(51:3) | 0.5196 | 0.3949 | 2.7949 | 2.3222 | 0.0068 |
| PA(10:0/19:0) | 0.6783 | 0.4746 | 3.5921 | 3.5417 | 0.0189 |
| PC(14:0/16:1(9Z)) | 12.1466 | 9.2236 | 62.361 | 61.4495 | 0.0199 |
| 25-Hydroxytachysterol3 | 0.0894 | 0.0834 | 0.4551 | 0.3793 | 0.0081 |
| CerP(d18:1/12:0) | 12.2274 | 12.7044 | 60.414 | 41.4471 | 0.0025 |
| Octadecanol | 6.3309 | 6.3619 | 30.05 | 28.4705 | 0.0192 |
| PC(38:7) | 0.6232 | 0.511 | 2.7654 | 2.1564 | 0.0068 |
| Palmitaldehyde | 17.6771 | 13.5223 | 74.823 | 61.5293 | 0.0102 |
| Hexyl heptanoate | 0.2627 | 0.2345 | 1.0832 | 0.9724 | 0.0183 |
| Perillic acid | 7.0925 | 6.8107 | 25.169 | 19.9423 | 0.0143 |
| PS(33:3) | 0.2288 | 0.1621 | 0.7956 | 0.5607 | 0.0066 |
| DG(40:2) | 0.4363 | 0.3662 | 1.5094 | 1.1243 | 0.0102 |
| Methyl linoleate | 14.6615 | 9.355 | 48.894 | 33.9512 | 0.0065 |
| LysoPE(0:0/16:0) | 77.9067 | 49.1045 | 254.76 | 189.556 | 0.0105 |
| TG(18:4/18:4/22:5) | 38.9224 | 55.4764 | 124.6 | 96.0658 | 0.0251 |
| 3-Hexenoic acid | 12.2265 | 7.8762 | 39.107 | 30.7764 | 0.0154 |
| PE(32:1) | 6.3614 | 4.4317 | 18.684 | 14.0517 | 0.0165 |
| 2-Nonenoic acid | 0.128 | 0.0938 | 0.3759 | 0.2635 | 0.0118 |
| LysoPE(20:1(11Z)/0:0) | 15.5527 | 11.8314 | 45.2 | 29.8558 | 0.0092 |
| Senecioic acid | 5.2296 | 4.0513 | 14.89 | 10.091 | 0.0116 |
| MG(0:0/18:1(11Z)/0:0) | 0.0387 | 0.0246 | 0.1092 | 0.0676 | 0.0062 |
| 5-oxo-Valeric acid | 9.2237 | 5.8193 | 23.966 | 14.2153 | 0.0071 |
| Palmitoyl Serinol | 0.188 | 0.0869 | 0.4845 | 0.3574 | 0.0201 |
| Acide colnelenique | 6.9857 | 4.6565 | 16.828 | 11.0852 | 0.0185 |
| DAG(32:1) | 0.0588 | 0.0523 | 0.1411 | 0.0879 | 0.0203 |
| Norecasantalic acid | 21.2216 | 13.4166 | 50.21 | 35.0955 | 0.0253 |
| LysoPA(18:0E) | 0.2206 | 0.1567 | 0.5158 | 0.2485 | 0.0052 |
| DG(15:0/0:0/20:4n3) | 41.436 | 26.2976 | 96.71 | 57.6488 | 0.0129 |
| LysoPE(0:0/18:1(9Z)) | 20.0086 | 13.2374 | 46.302 | 27.8765 | 0.0148 |
| Stearoyllactic acid | 0.0808 | 0.0363 | 0.1844 | 0.1125 | 0.0126 |
| GPC(22:5n6) | 9.0341 | 7.0644 | 19.942 | 12.0498 | 0.0238 |
| PA(40:8) | 0.3737 | 0.2089 | 0.7981 | 0.4136 | 0.0096 |
| PA(15:0/20:5) | 0.5279 | 0.2018 | 0.2705 | 0.1246 | 0.003 |
| DG(22:4n6/0:0/22:6n3) | 2.3678 | 1.2495 | 1.0935 | 0.9378 | 0.0189 |
| CL(52:0) | 0.1698 | 0.1092 | 0.0766 | 0.0264 | 0.0172 |
| Inodxyl glucuronide | 21.861 | 13.1881 | 9.8027 | 6.1001 | 0.0172 |
| AS 1-1 | 737.6 | 502.26 | 325.361 | 223.3993 | 0.0291 |
| TG(42:1) | 0.6328 | 0.3823 | 0.272 | 0.1487 | 0.0123 |
| DG(20:0/22:0/0:0) | 0.5067 | 0.2637 | 0.2171 | 0.119 | 0.0054 |
| Cheirotoxol | 69.63 | 38.4134 | 29.7363 | 25.4618 | 0.0135 |
| MG(i-17:0/0:0/0:0) | 20.019 | 13.5152 | 8.549 | 6.1 | 0.0249 |
| TAG(72:3) | 26.074 | 17.5861 | 11.1212 | 5.3308 | 0.0191 |
| LysoPA(0:0/16:0) | 0.3412 | 0.2025 | 0.1452 | 0.1074 | 0.0145 |
| 4-Hydroxyestradiol | 26.578 | 17.1182 | 11.29 | 7.1182 | 0.0178 |
| PE(34:3) | 185.79 | 126.7056 | 78.48 | 54.4065 | 0.0242 |
| MG(O-18:0/0:0/0:0) | 31.751 | 21.3306 | 13.0737 | 8.0655 | 0.0185 |
| CL(18:0/18:0/18:0/18:0) | 41.796 | 29.654 | 17.134 | 11.0553 | 0.024 |
| Nonyl octanoate | 6.0674 | 3.6522 | 2.472 | 1.6686 | 0.0111 |
| PS(15:0/22:0) | 16.2593 | 12.5137 | 6.6167 | 3.3928 | 0.0303 |
| PG(i-13:0/a-15:0) | 41.195 | 24.3468 | 16.671 | 12.2382 | 0.0107 |
| Decyl acetate | 58.209 | 47.3863 | 23.3 | 15.7291 | 0.0402 |
| Fusidic Acid | 1.6087 | 0.9962 | 0.6356 | 0.4968 | 0.0128 |
| Linalyl anthranilate | 0.2562 | 0.1169 | 0.0999 | 0.0834 | 0.0029 |
| LPE(20:1) | 0.5305 | 0.2799 | 0.2068 | 0.1509 | 0.0048 |
| Cholesterol sulfate | 7.092 | 4.1813 | 2.7169 | 1.9296 | 0.0076 |
| TG(24:0/24:0/24:0) | 40.734 | 25.2335 | 15.4402 | 10.2772 | 0.0088 |
| PE(14:1/20:5) | 323.37 | 228.2965 | 122.288 | 73.4688 | 0.0162 |
| Bisnorcholic acid | 2.6105 | 1.5655 | 0.9815 | 0.4319 | 0.0053 |
| PA(44:7) | 0.4474 | 0.3027 | 0.1673 | 0.0844 | 0.0114 |
| N-Stearoylsphingosine | 8.318 | 5.8249 | 3.1013 | 2.4563 | 0.0177 |
| DG(10:0/a-25:0/0:0) | 0.8578 | 0.4488 | 0.3186 | 0.2262 | 0.0032 |
| Armillaramide | 6.676 | 3.4099 | 2.4482 | 1.9042 | 0.003 |
| LysoPE(14:0/0:0) | 50.25 | 31.3451 | 18.3855 | 11.0982 | 0.0072 |
| 9,10,13-TriHOME | 1.2324 | 0.6335 | 0.4496 | 0.2652 | 0.002 |
| CE(15M5) | 0.8021 | 0.5575 | 0.2912 | 0.2271 | 0.0152 |
| Deoxycholic acid | 8.1207 | 5.0564 | 2.9357 | 3.0844 | 0.0127 |
| Dehydrocyanaropicrin | 0.247 | 0.134 | 0.0888 | 0.0481 | 0.0025 |
| Arjunolic acid | 2.0839 | 1.2284 | 0.7478 | 0.5439 | 0.0056 |
| SM(d18:1/15:0) | 15.276 | 10.7329 | 5.476 | 4.3631 | 0.0155 |
| CPA(18:1(11Z)/0:0) | 3.8824 | 2.3843 | 1.3913 | 0.8519 | 0.006 |
| PA(32:5) | 355.93 | 294.6982 | 127.36 | 76.9989 | 0.029 |
| PA(34:1) | 1.4576 | 0.8453 | 0.5192 | 0.4237 | 0.0057 |
| TAG(20:2/22:1/22:6) | 214.09 | 170.398 | 75.358 | 48.5183 | 0.0234 |
| GPG(18:2(9Z,12Z)) | 31.68 | 17.0309 | 11.0935 | 7.9561 | 0.0028 |
| DG(34:2) | 22.465 | 13.4924 | 7.843 | 5.914 | 0.0057 |
| PA(22:5/24:1) | 28.41 | 14.6976 | 9.7431 | 7.4469 | 0.0021 |
| PA(8:0/14:0) | 17.595 | 10.6592 | 6.0067 | 5.207 | 0.0063 |
| CE(19:0) | 3.2028 | 1.6804 | 1.0895 | 1.0891 | 0.0037 |
| TG(44:1) | 0.4932 | 0.3556 | 0.166 | 0.1095 | 0.0123 |
| DG(34:0) | 17.9 | 12.9286 | 6.0137 | 4.3144 | 0.013 |
| Thujyl alcohol | 26.106 | 12.7485 | 8.5716 | 10.1441 | 0.0032 |
| LysoPA(21:0/0:0) | 0.8903 | 0.4792 | 0.2885 | 0.2516 | 0.0025 |
| Pentyl heptanoate | 15.6724 | 12.5225 | 5.0718 | 5.0343 | 0.0231 |
| Epilubimin | 5.9446 | 5.0487 | 1.8989 | 1.3887 | 0.0251 |
| Acetic acid | 0.5842 | 0.4032 | 0.1858 | 0.1413 | 0.0086 |
| FAHFA(16:1(9Z)/5-O-18:0) | 3.2566 | 1.6908 | 1.0311 | 0.7975 | 0.0014 |
| Butyric acid | 0.1649 | 0.1067 | 0.0518 | 0.0516 | 0.0074 |
| PGP(16:1(9Z)/16:1(9Z)) | 24.664 | 17.3633 | 7.7073 | 4.9105 | 0.0082 |
| Pipecuronium | 112.69 | 60.053 | 35.2062 | 51.6074 | 0.0063 |
| Sciadonic acid | 0.4571 | 0.4088 | 0.1425 | 0.1011 | 0.0296 |
| Rosmic acid | 32.775 | 22.7215 | 10.1968 | 10.3561 | 0.0104 |
| Camelledionol | 1.2648 | 0.7492 | 0.3854 | 0.3947 | 0.0041 |
| LysoPI(18:1(9Z)/0:0) | 1.8504 | 1.5194 | 0.5634 | 0.2965 | 0.017 |
| PC(18:1) | 16.201 | 11.0415 | 4.8988 | 3.5855 | 0.0065 |
| PA(39:1) | 42.82 | 27.8379 | 12.8801 | 9.7103 | 0.0048 |
| LysoPC(P-18:0/0:0) | 194.19 | 148.6435 | 57.9203 | 62.9116 | 0.0156 |
| LysoPG(16:0/0:0) | 0.0568 | 0.0339 | 0.0168 | 0.0141 | 0.0029 |
| Pisumic acid | 32.476 | 28.3367 | 9.567 | 3.7642 | 0.0208 |
| 1,24,25-Trihydroxyvitamin D2 | 544.49 | 492.4501 | 159.31 | 154.9756 | 0.0298 |
| PIP(16:1(9Z)/18:0) | 4.6162 | 3.325 | 1.3439 | 1.0963 | 0.0085 |
| PI(16:0/20:2(11Z,14Z)) | 1.7023 | 1.4663 | 0.491 | 0.3778 | 0.021 |
| 15-Epi-lipoxin A4 | 23.007 | 15.2641 | 6.5825 | 5.3003 | 0.0048 |
| LysoPS(18:1(9Z)/0:0) | 3.9438 | 3.4616 | 1.1185 | 0.8213 | 0.0218 |
| PGP(16:0/18:0) | 0.2799 | 0.1818 | 0.0792 | 0.0634 | 0.004 |
| LPC(p-16:0/0:0) | 18.82 | 14.6107 | 5.2671 | 4.4319 | 0.0117 |
| DG(15:0/0:0/16:1n7) | 0.7717 | 0.5525 | 0.2154 | 0.1719 | 0.007 |
| Sorbitan stearate | 25.272 | 19.0074 | 6.9329 | 5.8872 | 0.0093 |
| Pubesenolide | 55.41 | 39.8945 | 15.1563 | 15.9324 | 0.0083 |
| LPA(22:4) | 87.7 | 59.2804 | 23.5247 | 19.7206 | 0.0045 |
| Tetracosanoic acid | 82.99 | 50.599 | 22.1588 | 16.8575 | 0.002 |
| 3beta-3-Lupanol | 2.189 | 1.5105 | 0.582 | 0.4919 | 0.005 |
| Sterol | 0.3971 | 0.2743 | 0.1022 | 0.0727 | 0.0041 |
| PA(10:0/8:0) | 22.805 | 15.7599 | 5.8551 | 4.3122 | 0.0042 |
| Prednisone | 40.733 | 25.3245 | 10.4409 | 8.2243 | 0.0021 |
| LysoPE(20:0/0:0) | 87.296 | 67.8186 | 22.258 | 18.0592 | 0.0089 |
| PA(42:1) | 1782.6 | 1168.214 | 453.411 | 367.4364 | 0.003 |
| PGP(a-13:0/i-22:0) | 33.74 | 24.3974 | 8.4558 | 8.0189 | 0.006 |
| Methyl dihydrophaseate | 6.2933 | 6.2055 | 1.5772 | 1.4107 | 0.0308 |
| alpha-Peroxyachifolide | 25.1 | 12.6699 | 6.2724 | 4.8213 | 0.0004 |
| PC(14:0/P-18:0) | 0.1822 | 0.1182 | 0.0452 | 0.0323 | 0.0024 |
| 2-Ethylsuberic acid | 0.1088 | 0.0771 | 0.0267 | 0.0148 | 0.0039 |
| Propionic acid | 1.0291 | 0.6643 | 0.2504 | 0.1535 | 0.002 |
| PA(16:0/18:2(9Z,12Z)) | 21.818 | 17.4502 | 5.233 | 5.9717 | 0.0108 |
| alpha-Irone | 1.3418 | 1.1242 | 0.3082 | 0.2707 | 0.0112 |
| PC(20:5/P-18:1) | 5.287 | 3.0503 | 1.1711 | 0.9679 | 0.0007 |
| Avenoleic acid | 32.775 | 28.8532 | 7.2519 | 7.7234 | 0.0146 |
| PGP(16:0/16:1(9Z)) | 50.5 | 29.3088 | 11.1324 | 9.1167 | 0.0007 |
| Carnosic acid | 210.16 | 202.1272 | 44.1171 | 39.3186 | 0.0201 |
| LysoPC(18:1(11Z)/0:0) | 2.6664 | 1.407 | 0.5556 | 0.5096 | 0.0003 |
| 7-Sulfocholic acid | 1.0051 | 0.6268 | 0.1988 | 0.1905 | 0.0011 |
| LysoPC(18:1(11Z)) | 22.119 | 17.3022 | 4.3198 | 3.3255 | 0.005 |
| PA(35:2) | 39.525 | 33.6505 | 7.5898 | 5.1648 | 0.0083 |
| DAG(40:4) | 4.0939 | 3.696 | 0.7839 | 0.9723 | 0.0135 |
| MG(0:0/14:1(9Z)/0:0) | 0.5902 | 0.4393 | 0.1027 | 0.0976 | 0.003 |
| Prenyl glucoside | 27.035 | 23.247 | 3.9696 | 4.2692 | 0.0064 |
| Isodigeranyl | 0.3567 | 0.3979 | 0.0516 | 0.0603 | 0.0276 |
| MG(0:0/18:2(9Z,12Z)/0:0) | 30.9907 | 37.6579 | 4.4115 | 3.1515 | 0.0392 |
| PA(8:0/i-15:0) | 22.4077 | 24.5778 | 2.6833 | 2.4466 | 0.0212 |
| PA(10:0/8:0) | 1.6647 | 1.9416 | 0.1616 | 0.1953 | 0.0255 |
| LPE(0:0/18:4n3) | 35.1596 | 39.7668 | 3.216 | 3.5281 | 0.0209 |

**Table S5 Differential liver metabolites between the two groups**

| **Metabolites** | **CM** |  | **DM** |  | **p** |
| --- | --- | --- | --- | --- | --- |
|  | Mean | SD | Mean | SD |  |
| TG(16:0/18:2/18:2) | 1175.622 | 1718.615 | 5778.039 | 6442.547 | 0.0425 |
| CerG2GNAc1(d36:2) | 0.4722 | 0.2364 | 1.341 | 0.8095 | 0.0066 |
| CerG2GNAc1(d36:1) | 0.7067 | 0.5971 | 1.9577 | 0.8263 | 0.0011 |
| TG(62:7e) | 2.2321 | 1.1325 | 5.6057 | 2.8843 | 0.0029 |
| DG(30:3e) | 5.2244 | 5.6798 | 12.9075 | 9.2067 | 0.0375 |
| TG(16:0/8:0/18:1) | 9.2711 | 13.4725 | 22.0007 | 11.1222 | 0.0333 |
| MePC(41:4e) | 13.0445 | 7.4309 | 30.1825 | 12.1 | 0.0013 |
| TG(70:4) | 0.8931 | 0.6806 | 2.0586 | 1.2191 | 0.0166 |
| DG(35:1e) | 4.3015 | 3.0426 | 9.4847 | 5.4462 | 0.0171 |
| ChE(18:1) | 5.3531 | 2.6685 | 11.6075 | 6.3169 | 0.0099 |
| DG(33:1e) | 9.2862 | 6.6378 | 19.4568 | 10.5801 | 0.0191 |
| TG(17:0/18:1/18:1) | 395.8867 | 279.5049 | 813.3348 | 454.1711 | 0.0235 |
| TG(28:0/18:1/24:2) | 3.2074 | 2.8399 | 6.3425 | 3.5215 | 0.0418 |
| TG(16:0/16:1/18:2) | 2181.851 | 1569.857 | 4302.815 | 2326.242 | 0.028 |
| PC(20:0e/22:5) | 2.5662 | 1.6963 | 5.0417 | 1.8331 | 0.0057 |
| TG(20:0e/18:1/22:6) | 3.7853 | 1.6049 | 7.3649 | 4.0062 | 0.0172 |
| DG(33:0e) | 12.2262 | 8.628 | 23.3952 | 14.1336 | 0.047 |
| TG(18:1/18:2/20:4) | 121.2025 | 97.398 | 230.9386 | 102.0414 | 0.0242 |
| TG(16:0/17:1/18:1) | 505.2514 | 387.365 | 960.7751 | 554.3023 | 0.0472 |
| DG(34:5e) | 58.1812 | 39.0266 | 110.5577 | 57.9495 | 0.0291 |
| TG(28:1/16:0/24:2) | 3.4282 | 2.685 | 6.4619 | 3.4645 | 0.042 |
| TG(19:1/16:0/18:2) | 219.7629 | 139.9542 | 409.1694 | 200.8199 | 0.0249 |
| PE(42:5e) | 12.8523 | 4.1079 | 23.7097 | 8.2207 | 0.0015 |
| PIP(52:1) | 8.0536 | 3.7508 | 14.7877 | 7.9142 | 0.0257 |
| LPE(18:1) | 69.3261 | 31.3767 | 125.1994 | 58.6973 | 0.0161 |
| TG(16:1/16:1/18:2) | 251.816 | 191.0614 | 450.7653 | 226.8785 | 0.0481 |
| DG(34:1e) | 440.3685 | 274.7464 | 772.4679 | 335.8195 | 0.0263 |
| TG(16:0/18:1/22:6) | 997.8173 | 697.3333 | 1748.007 | 836.3994 | 0.0429 |
| TG(16:0/14:4/16:0) | 9.024 | 6.2901 | 15.7153 | 7.5693 | 0.0454 |
| TG(16:0/18:1/18:3) | 4562.003 | 3017.926 | 7851.757 | 3714.242 | 0.0433 |
| TG(18:0/17:0/18:3) | 52.5515 | 29.2131 | 88.7658 | 40.0132 | 0.0329 |
| TG(16:0/18:1/18:1) | 18837.66 | 12000.57 | 31551.87 | 13202.65 | 0.0369 |
| TG(52:3) | 858.0992 | 529.1958 | 1427.33 | 631.2591 | 0.0423 |
| LPE(16:0) | 185.8364 | 66.6414 | 306.4344 | 101.8395 | 0.0057 |
| TG(16:0/18:1/18:2) | 14321.76 | 8091.475 | 23590.93 | 8751.821 | 0.0243 |
| LPE(20:1) | 7.231 | 3.1216 | 11.8442 | 4.7178 | 0.0189 |
| DG(36:5e) | 258.3805 | 143.4485 | 423.0978 | 173.4545 | 0.0327 |
| TG(18:1/18:1/18:2) | 2426.387 | 1138.051 | 3893.249 | 1508.091 | 0.0245 |
| PC(40:3e) | 38.9151 | 11.4873 | 61.9311 | 20.6293 | 0.0064 |
| CerG2GNAc1(d34:2) | 1.1606 | 0.4486 | 1.8392 | 0.6108 | 0.0111 |
| LPE(16:2e) | 10.8835 | 3.0394 | 17.2043 | 5.1324 | 0.0036 |
| LPE(18:0) | 346.7497 | 107.1235 | 547.7435 | 148.9212 | 0.0028 |
| PI(17:0/20:3) | 1.7451 | 0.8311 | 2.7381 | 1.2288 | 0.0485 |
| PC(36:0e) | 25.433 | 6.1998 | 39.1863 | 12.1876 | 0.0052 |
| LPE(20:0) | 1.8694 | 0.931 | 2.879 | 0.8536 | 0.0211 |
| CerG2GNAc1(d34:1) | 1.8448 | 0.937 | 2.829 | 1.099 | 0.045 |
| LPC(13:0) | 39.4866 | 12.5788 | 60.4266 | 16.7319 | 0.0054 |
| TG(19:0/19:0/20:3) | 54.1238 | 21.9592 | 82.0539 | 32.4363 | 0.0368 |
| LPI(18:0) | 6.3637 | 3.0839 | 9.558 | 2.1736 | 0.0154 |
| PS(20:5/18:2) | 15.4198 | 3.6394 | 22.9703 | 6.152 | 0.0036 |
| TG(18:1/18:1/18:1) | 2921.713 | 1265.997 | 4318.922 | 1413.192 | 0.0317 |
| SM(d39:0) | 4.8584 | 2.0987 | 7.178 | 1.3258 | 0.0097 |
| LPI(20:4) | 3.7504 | 1.4826 | 5.486 | 1.4364 | 0.016 |
| PE(20:0e/18:2) | 12.535 | 2.8623 | 17.9398 | 5.1893 | 0.0099 |
| PE(20:1e) | 11.7935 | 4.9084 | 16.8204 | 4.0328 | 0.0222 |
| PC(38:2e) | 45.9618 | 10.6224 | 65.5044 | 17.2241 | 0.0068 |
| LPE(18:2e) | 16.0617 | 5.2154 | 22.864 | 6.5515 | 0.0193 |
| PE(20:1e/18:2) | 21.0697 | 4.4279 | 29.9392 | 8.0142 | 0.0067 |
| BiotinylPE(16:4) | 5.7121 | 1.9451 | 8.0868 | 1.8978 | 0.0128 |
| TG(20:0e/16:0/18:1) | 29.4598 | 8.6328 | 41.3258 | 14.5422 | 0.0396 |
| LPC(18:1) | 168.5926 | 72.9304 | 234.7121 | 53.934 | 0.0333 |
| PC(42:5e) | 54.5093 | 10.5142 | 75.7346 | 18.7362 | 0.0059 |
| PE(20:0p/22:5) | 63.347 | 13.8149 | 87.4629 | 21.758 | 0.0084 |
| MePC(14:0e) | 57.9197 | 15.781 | 78.2296 | 15.018 | 0.0086 |
| SM(d18:1/24:2) | 45.7643 | 14.2507 | 60.4245 | 10.7224 | 0.0181 |
| SM(d43:3) | 10.9132 | 2.6593 | 14.2663 | 3.3979 | 0.0244 |
| SM(d43:4) | 658.0162 | 142.0533 | 860.0218 | 206.7688 | 0.0203 |
| PE(20:0p/18:2) | 34.4368 | 6.2152 | 45.0069 | 14.5303 | 0.0486 |
| PE(18:0p/20:1) | 16.0141 | 3.8409 | 20.8946 | 5.1485 | 0.0273 |
| PE(16:0/22:5) | 485.6347 | 150.1547 | 631.1444 | 70.2834 | 0.0125 |
| PE(18:0e/18:2) | 49.1978 | 10.6122 | 63.4552 | 16.6757 | 0.0349 |
| SM(d41:0) | 10.1827 | 2.1813 | 13.0691 | 3.1227 | 0.0276 |
| LPE(20:0e) | 1.7889 | 0.4118 | 2.2959 | 0.5779 | 0.0365 |
| PE(20:0e/22:6) | 36.1442 | 7.6202 | 45.3615 | 9.3005 | 0.0261 |
| LPC(18:2e) | 8.8781 | 2.2538 | 11.0885 | 2.2033 | 0.0397 |
| PC(20:0e/22:6) | 5.1399 | 1.0036 | 6.4139 | 1.523 | 0.0404 |
| LPC(18:0) | 460.4508 | 75.9469 | 574.5309 | 99.1496 | 0.0098 |
| LdMePE(18:0) | 36.0958 | 5.8105 | 44.1485 | 7.5165 | 0.0153 |
| PC(42:6e) | 77.4867 | 16.4065 | 94.6996 | 18.0203 | 0.0384 |
| CL(20:5/18:1/20:1/18:2) | 1.229 | 0.2184 | 1.501 | 0.2822 | 0.0269 |
| PC(20:0e/20:4) | 6.1926 | 1.0062 | 7.5446 | 1.2966 | 0.0179 |
| PC(40:2e) | 117.8834 | 21.3741 | 142.3129 | 20.732 | 0.0183 |
| PE(16:0/20:5) | 137.157 | 20.1883 | 161.1231 | 29.6565 | 0.0489 |
| PC(18:0/18:1) | 1484.822 | 218.7792 | 1730.134 | 285.6273 | 0.0449 |
| PC(33:3) | 1176.574 | 196.3331 | 1350.861 | 152.6808 | 0.0398 |
| PC(36:2e) | 223.234 | 30.5283 | 252.8271 | 27.1305 | 0.0342 |
| Co(Q8) | 797.69 | 87.6868 | 883.1441 | 69.9939 | 0.027 |
| PS(42:7) | 35.5144 | 3.1475 | 32.0466 | 3.3299 | 0.0278 |
| PC(34:2) | 205228.4 | 23278.73 | 184959.3 | 19677.25 | 0.0498 |
| Hex1Cer(d19:1/22:6) | 2124.454 | 258.4039 | 1887.912 | 193.5314 | 0.0325 |
| Cer(t18:0/24:3) | 1097.142 | 91.477 | 974.8581 | 137.6961 | 0.0311 |
| Cer(d18:1/24:3) | 1098.435 | 105.6116 | 969.1889 | 118.6118 | 0.0191 |
| PS(18:2/18:2) | 316.3898 | 31.7014 | 276.5477 | 47.9243 | 0.0417 |
| CL(18:3/18:2/18:1/16:0) | 3.2596 | 0.4709 | 2.8422 | 0.3594 | 0.0389 |
| PG(17:0/17:0) | 119.2668 | 14.2625 | 103.9727 | 11.1054 | 0.0154 |
| OAHFA(47:4) | 215.8253 | 19.1566 | 188.0078 | 22.9837 | 0.0088 |
| PE(16:0p/20:4) | 1336.699 | 214.0958 | 1153.64 | 128.8337 | 0.0325 |
| PC(16:0/18:2) | 9934.248 | 748.8445 | 8522.422 | 1134.817 | 0.0041 |
| PE(18:2e/20:4) | 231.3318 | 29.1346 | 197.8055 | 20.3815 | 0.008 |
| PC(36:0) | 214.7099 | 35.5066 | 183.5876 | 18.9071 | 0.0249 |
| PE(18:0e/20:4) | 168.4714 | 25.4761 | 144.0128 | 19.4248 | 0.0266 |
| DG(16:0/16:0) | 36.8114 | 5.7227 | 31.3071 | 4.6746 | 0.03 |
| PC(36:2) | 56085.34 | 10907.22 | 47589.96 | 6525.872 | 0.0488 |
| CL(18:3/20:4/20:4/18:2) | 6.4859 | 0.7538 | 5.4827 | 1.036 | 0.0234 |
| PI(18:0/20:4) | 661.7414 | 98.2356 | 550.7273 | 69.4126 | 0.0092 |
| PE(18:1p/22:6) | 174.3384 | 36.9205 | 144.879 | 20.0259 | 0.0397 |
| PE(20:4/21:1) | 42.0116 | 5.8128 | 34.7676 | 4.4209 | 0.0057 |
| PC(20:1/14:3) | 6.8785 | 1.0878 | 5.6466 | 1.0012 | 0.0168 |
| PS(17:1/20:2) | 6.8828 | 1.0881 | 5.6481 | 1.0013 | 0.0166 |
| PG(19:1/16:0) | 7.9221 | 1.076 | 6.4602 | 0.7008 | 0.002 |
| TG(19:1/17:1/19:1) | 6.0894 | 1.4376 | 4.9109 | 0.8427 | 0.0382 |
| PC(18:0/18:0) | 17.7939 | 3.2216 | 14.1476 | 1.3846 | 0.0041 |
| PE(17:0/20:4) | 189.2998 | 50.1204 | 150.4148 | 21.7829 | 0.0372 |
| PE(18:1e/22:4) | 77.4652 | 10.9688 | 61.4942 | 10.081 | 0.0033 |
| PIP(33:1) | 30.0005 | 6.5675 | 23.7917 | 4.329 | 0.0225 |
| PE(16:0p/22:5) | 984.3231 | 161.2929 | 780.5147 | 97.4411 | 0.0031 |
| PC(18:0e/20:4) | 22.9035 | 4.3418 | 18.061 | 3.2058 | 0.0109 |
| MGDG(18:0/20:3) | 93.9385 | 12.6334 | 74.0332 | 7.2776 | 0.0004 |
| DG(18:0/16:0) | 50.2773 | 12.7036 | 39.5403 | 5.2093 | 0.0236 |
| PC(35:0) | 58.9675 | 13.6624 | 46.3036 | 4.5573 | 0.0123 |
| TG(18:0/16:0/18:0) | 1510.529 | 409.0888 | 1179.28 | 92.484 | 0.0224 |
| PE(18:0p/22:6) | 286.0668 | 47.0546 | 223.0505 | 34.0259 | 0.003 |
| SM(d35:1) | 6.9598 | 1.6151 | 5.4165 | 0.9521 | 0.018 |
| MePC(34:4e) | 273.6076 | 39.7918 | 212.787 | 38.2055 | 0.0026 |
| Cer(d18:0/22:0) | 510.3425 | 140.4678 | 396.2205 | 53.9404 | 0.0275 |
| Cer(d18:2/24:2) | 21.8341 | 5.7026 | 16.9159 | 2.2987 | 0.021 |
| PC(16:0/16:0) | 1161.469 | 190.8834 | 898.8974 | 149.0741 | 0.003 |
| PC(18:1e/20:4) | 49.3884 | 6.4578 | 38.1496 | 8.5803 | 0.0039 |
| PC(16:0/20:4) | 5450.124 | 817.9254 | 4209.042 | 881.2601 | 0.0043 |
| PS(42:7e) | 17.5862 | 2.1232 | 13.5344 | 2.3038 | 0.0007 |
| CL(18:2/16:0/16:0/18:1) | 9.8263 | 2.2517 | 7.5616 | 1.3344 | 0.0136 |
| PC(16:0e/20:4) | 48.5418 | 10.6827 | 37.1703 | 11.3955 | 0.0335 |
| Cer(d36:4) | 2.2782 | 0.4171 | 1.743 | 0.2562 | 0.0028 |
| PE(40:9) | 264.1915 | 73.6423 | 201.7289 | 32.3145 | 0.0244 |
| PI(18:0/16:1) | 269.9466 | 84.8626 | 206.1035 | 39.1156 | 0.0445 |
| PE(19:1/20:4) | 45.8963 | 9.2373 | 35.0019 | 8.0623 | 0.0116 |
| PC(34:0) | 2750.712 | 414.3018 | 2090.455 | 285.0956 | 0.0006 |
| TG(18:0/18:0/20:3) | 249.8884 | 81.3619 | 189.3406 | 33.6196 | 0.0432 |
| PC(29:0) | 11.4936 | 2.2496 | 8.691 | 3.0245 | 0.0303 |
| PI(16:0/22:6) | 28.731 | 5.9662 | 21.6533 | 3.951 | 0.0058 |
| BiotinylPE(27:3) | 9.9269 | 2.6688 | 7.4648 | 1.4655 | 0.0198 |
| PA(24:2/22:6) | 2555.345 | 424.8947 | 1921.491 | 591.6242 | 0.0131 |
| SM(d42:3) | 209.4662 | 55.7967 | 157.4432 | 29.3177 | 0.0177 |
| PI(17:0/20:4) | 14.3267 | 2.707 | 10.7667 | 1.9367 | 0.0033 |
| PE(20:0p/20:4) | 108.6145 | 15.7373 | 81.4365 | 13.7756 | 0.0007 |
| PC(40:0) | 19.5885 | 5.9851 | 14.6849 | 2.1241 | 0.0252 |
| Cer(d29:0) | 4.1632 | 1.4725 | 3.112 | 0.4427 | 0.0443 |
| TG(18:0/18:0/18:0) | 553.2493 | 171.941 | 412.8949 | 53.4788 | 0.024 |
| PS(18:0/20:3) | 36.2076 | 6.7031 | 26.9835 | 4.7611 | 0.0023 |
| PC(18:0/16:0) | 231.9898 | 39.0498 | 172.3619 | 25.2213 | 0.0007 |
| DG(36:2e) | 105.5751 | 36.8422 | 78.4392 | 12.1532 | 0.0401 |
| PS(40:7e) | 72.7515 | 14.8988 | 54.0244 | 11.0223 | 0.005 |
| MePC(32:5e) | 104.4033 | 19.6682 | 77.4739 | 14.0103 | 0.0024 |
| TG(25:0/16:0/16:0) | 28.595 | 8.4035 | 21.2134 | 3.6414 | 0.0202 |
| PC(31:0) | 514.1503 | 135.8514 | 379.9916 | 64.914 | 0.0114 |
| PE(20:0/18:1) | 451.088 | 72.7299 | 331.6651 | 65.3862 | 0.0011 |
| PC(32:0) | 12582.1 | 3168.073 | 9231.681 | 2097.057 | 0.0121 |
| PG(38:6) | 4.3353 | 1.3429 | 3.1787 | 1.0983 | 0.0493 |
| Cer(d30:3) | 17.0098 | 5.76 | 12.4691 | 2.7196 | 0.0369 |
| Cer(d30:0) | 557.2478 | 212.7317 | 406.7203 | 71.38 | 0.048 |
| TG(16:0e/16:0/18:0) | 4.6911 | 1.6324 | 3.4111 | 0.7079 | 0.0354 |
| TG(18:0/18:0/19:0) | 64.9667 | 22.8171 | 47.1531 | 8.4937 | 0.0327 |
| PS(18:0/22:5) | 17.1403 | 4.0906 | 12.4396 | 3.0081 | 0.009 |
| PI(38:6) | 7.3003 | 2.2082 | 5.2972 | 1.2838 | 0.0233 |
| MePC(34:5e) | 104.7662 | 27.5974 | 76.0069 | 16.1847 | 0.0108 |
| TG(12:0/10:3/10:4) | 10.2353 | 3.5368 | 7.4238 | 1.0337 | 0.0267 |
| PI(18:0/22:6) | 47.9255 | 7.9232 | 34.7516 | 6.8925 | 0.0009 |
| Cer(t20:0/26:0) | 2.015 | 0.7113 | 1.4597 | 0.4108 | 0.0465 |
| SM(d36:1) | 481.7455 | 154.516 | 347.4342 | 70.9449 | 0.0224 |
| TG(15:0/16:0/18:3) | 21.0646 | 5.3188 | 15.166 | 4.1444 | 0.0127 |
| PS(20:0/22:6) | 46.7415 | 5.8632 | 33.6001 | 7.4124 | 0.0003 |
| Cer(t18:0/18:0+O) | 29.9697 | 11.4776 | 21.4568 | 4.1511 | 0.0407 |
| PS(18:0/18:2) | 35.7998 | 8.2143 | 25.6097 | 4.2818 | 0.0027 |
| TG(15:0/14:0/16:0) | 30.6551 | 7.071 | 21.833 | 8.3824 | 0.0204 |
| Cer(t18:0/20:0+O) | 23.982 | 9.5175 | 17.0219 | 3.221 | 0.0419 |
| DG(32:1e) | 437.1154 | 144.722 | 310.1655 | 64.9164 | 0.0209 |
| DG(39:2) | 44.6964 | 12.9965 | 31.6387 | 7.0298 | 0.012 |
| PE(18:2e/20:3) | 703.1233 | 248.9351 | 497.0681 | 84.7636 | 0.0234 |
| Hex2Cer(d42:1) | 6.6504 | 1.1692 | 4.6951 | 1.3867 | 0.0031 |
| AEA(16:0) | 50.6421 | 13.868 | 35.6925 | 6.9905 | 0.007 |
| LBPA(16:0/18:2) | 63.9507 | 24.4031 | 45.0076 | 12.6837 | 0.0429 |
| Hex2Cer(d41:1) | 2.5579 | 0.7984 | 1.7895 | 0.713 | 0.0357 |
| PE(36:4) | 51.1355 | 11.5976 | 35.6835 | 13.0594 | 0.0119 |
| PC(15:0/20:4) | 10.6641 | 3.8498 | 7.4283 | 2.4655 | 0.0381 |
| PI(34:2) | 40.8583 | 10.1133 | 28.3559 | 7.2758 | 0.0053 |
| Cer(d16:0/20:0+O) | 92.3243 | 36.7739 | 64.0219 | 12.7827 | 0.0337 |
| PC(16:1e/20:4) | 21.1367 | 6.0855 | 14.6529 | 4.3529 | 0.0134 |
| CerG2GNAc1(d43:2) | 5.7628 | 2.1753 | 3.9927 | 1.3239 | 0.0413 |
| Hex2Cer(d18:1/24:1) | 20.0596 | 5.693 | 13.8617 | 2.4202 | 0.0053 |
| PS(38:4) | 340.9015 | 71.938 | 235.4159 | 128.2517 | 0.0358 |
| Cer(m36:3+2O) | 16.1238 | 7.0114 | 11.093 | 1.7291 | 0.0409 |
| PI(18:3e/18:2) | 8.3348 | 3.4041 | 5.6866 | 1.6138 | 0.0393 |
| PE(18:0p/20:4) | 364.7231 | 72.0536 | 248.6328 | 44.1051 | 0.0004 |
| PE(41:4e) | 48.2321 | 16.4295 | 32.7836 | 10.4441 | 0.0219 |
| PI(16:0/18:2) | 90.783 | 23.3095 | 61.6239 | 14.5222 | 0.0035 |
| PS(20:4/20:4) | 1.6938 | 0.5245 | 1.1475 | 0.4084 | 0.0181 |
| TG(18:0e/11:3/11:3) | 41.182 | 16.9379 | 27.8767 | 4.7049 | 0.0278 |
| PS(18:0/20:4) | 313.9917 | 71.5063 | 210.8855 | 33.2363 | 0.0006 |
| PC(20:0/20:3) | 39.5197 | 14.0901 | 26.3926 | 11.2823 | 0.0336 |
| AcCa(24:0) | 14.2482 | 4.5509 | 9.4736 | 2.6883 | 0.0105 |
| SPH(d18:0) | 1604.359 | 683.1601 | 1058.47 | 251.196 | 0.0291 |
| PI(18:0/18:2) | 108.8565 | 28.3719 | 71.8135 | 15.9543 | 0.0021 |
| PIP(40:9e) | 5.1451 | 1.1154 | 3.3861 | 1.0127 | 0.0017 |
| PS(16:0/22:6) | 52.3627 | 10.8606 | 34.4113 | 11.1552 | 0.0018 |
| PC(33:0) | 370.5932 | 72.8994 | 243.4534 | 49.1851 | 0.0002 |
| PG(16:0/20:2) | 40.0083 | 14.7581 | 26.1071 | 12.7153 | 0.0367 |
| Hex2Cer(d42:2) | 12.3435 | 2.8735 | 7.9951 | 2.0789 | 0.0011 |
| Hex2Cer(d40:1) | 7.4277 | 1.868 | 4.8016 | 1.4428 | 0.0025 |
| PG(16:0/18:3) | 4.2755 | 1.8324 | 2.7569 | 1.1791 | 0.0408 |
| AcCa(20:4) | 265.723 | 107.744 | 169.7166 | 71.6644 | 0.0306 |
| PS(18:0/22:6) | 624.931 | 165.7573 | 398.2672 | 87.3777 | 0.0012 |
| PS(16:0/20:4) | 46.5334 | 11.5052 | 29.5565 | 6.9399 | 0.0008 |
| DG(38:5e) | 38.539 | 10.8283 | 24.1951 | 5.1892 | 0.0014 |
| SM(d44:6) | 333.1642 | 146.2 | 207.0762 | 59.9477 | 0.0212 |
| Cer(d20:0/24:0) | 8.725 | 2.2021 | 5.3785 | 1.6906 | 0.0013 |
| BiotinylPE(34:9) | 11.2066 | 5.0432 | 6.888 | 2.7018 | 0.0282 |
| PE(20:4e/17:1) | 23.9564 | 4.5997 | 14.7079 | 3.4629 | 0.0001 |
| SM(t34:1) | 72.4042 | 23.0347 | 44.2546 | 14.8737 | 0.0045 |
| PA(42:5e) | 5.5616 | 1.9609 | 3.3692 | 1.4083 | 0.0101 |
| LBPA(16:0/20:4) | 74.2187 | 39.356 | 44.6436 | 17.5088 | 0.0435 |
| phSM(d16:0/18:1) | 5.7498 | 2.0886 | 3.4557 | 1.4779 | 0.011 |
| PG(16:0/18:1) | 74.6476 | 38.9568 | 44.7891 | 18.2857 | 0.0416 |
| DG(38:4e) | 25.0029 | 7.1592 | 14.9728 | 4.9305 | 0.0018 |
| PIP(42:9e) | 3.1415 | 1.237 | 1.836 | 0.6029 | 0.0077 |
| BiotinylPE(36:9) | 9.0126 | 4.4436 | 5.2577 | 1.7115 | 0.0226 |
| PC(38:8e) | 63.9306 | 34.2729 | 37.1414 | 13.3434 | 0.0334 |
| PI(36:2) | 14.531 | 3.953 | 8.3167 | 2.1482 | 0.0004 |
| TG(16:1/13:0/18:2) | 8.0576 | 3.4755 | 4.5694 | 2.7871 | 0.0234 |
| Cer(d38:2) | 15.9289 | 8.8074 | 8.982 | 3.3431 | 0.0315 |
| PIP2(18:2/22:6) | 3.2945 | 2.0232 | 1.8473 | 0.7572 | 0.0483 |
| PIP2(20:2/18:2) | 1.8242 | 1.0399 | 1.0226 | 0.3324 | 0.0322 |
| AEA(18:0) | 33.9215 | 15.9081 | 18.6805 | 3.6684 | 0.0085 |
| MePC(37:4e) | 43.4486 | 22.8956 | 23.8838 | 14.0188 | 0.0333 |
| PC(40:7e) | 45.5536 | 26.4455 | 24.2854 | 13.4925 | 0.0361 |
| PS(18:0/22:4) | 22.8613 | 5.403 | 11.8594 | 5.9272 | 0.0004 |
| AcCa(20:3) | 21.6503 | 10.8975 | 11.1754 | 6.1387 | 0.0163 |
| DG(39:7) | 18.6125 | 10.8377 | 9.5428 | 3.2878 | 0.0209 |
| PC(42:9e) | 15.18 | 7.5825 | 7.7371 | 3.0047 | 0.0098 |
| PG(34:2) | 26.1942 | 16.0766 | 13.2876 | 4.6215 | 0.0253 |
| AcCa(22:6) | 48.9252 | 26.7277 | 24.7527 | 12.59 | 0.0186 |
| TG(18:1e/16:0/20:4) | 17.7405 | 5.9368 | 8.8973 | 6.143 | 0.0042 |
| AcCa(17:1) | 5.7357 | 3.2105 | 2.8316 | 1.1199 | 0.0146 |
| AcCa(24:2) | 8.6821 | 4.5443 | 4.118 | 1.107 | 0.0064 |
| PIP2(18:2/20:4) | 2.8934 | 1.9212 | 1.3133 | 0.6244 | 0.0322 |
| AcCa(18:2) | 253.6349 | 139.573 | 112.5157 | 68.5516 | 0.0102 |
| PIP(16:0/10:1) | 14.8389 | 3.9456 | 6.4034 | 3.4612 | 0.0001 |
| AcCa(10:3) | 2.3337 | 1.5772 | 1.0032 | 0.4267 | 0.0191 |
| AcCa(20:2) | 14.2564 | 8.6844 | 5.9682 | 4.6582 | 0.016 |
| AcCa(22:5) | 3.1157 | 2.0381 | 1.2891 | 0.7226 | 0.0156 |
| AcCa(22:4) | 4.235 | 2.693 | 1.7363 | 0.6586 | 0.0106 |
| Cer(d36:3+O) | 29.6086 | 25.3807 | 11.5054 | 4.3634 | 0.0393 |
| PIP(8:0/20:3) | 21.1291 | 7.466 | 7.6488 | 4.0959 | 0.0001 |
| PIP(10:0/20:3) | 11.0148 | 5.1025 | 3.6362 | 2.3667 | 0.0006 |
| AcCa(20:5) | 1.8716 | 1.4568 | 0.5349 | 0.5538 | 0.0143 |
| PIP(16:0/12:3) | 3.5514 | 1.9015 | 0.9522 | 1.0231 | 0.0013 |

***4.*** ***Differential neurotransmitters***

In total, 16 neurotransmitters were detected in this study. Finally, seven neurotransmitters were found to be significantly different between the two groups (Supplementary Table 6)

**Table S6 Differential neurotransmitters between the two groups**

| **Neurotransmitters** | **CM** |  | **DM** |  | **p** |
| --- | --- | --- | --- | --- | --- |
|  | Mean | SD | Mean | SD |  |
| Glu | 13367.93 | 2727.449 | 10989.26 | 1065.026 | 0.019 |
| Gln | 7779.472 | 1016.963 | 6726.344 | 639.7694 | 0.013 |
| GABA | 81413.5 | 7859.684 | 66971.47 | 5388.795 | 0.001 |
| Phe | 21066.09 | 2138.503 | 16650.21 | 4086.917 | 0.007 |
| PEA | 0.7512 | 0.0989 | 0.5462 | 0.157 | 0.003 |
| VMA | 0.147 | 0.0653 | 0.6432 | 0.5185 | 0.007 |
| NE | 2.346 | 1.057 | 1.2564 | 0.4694 | 0.008 |

Abbreviations: Glu, glutamic acid; Gln, glutamine; GABA, gamma-aminobutyric acid; Phe, L-phenylalanine; PEA, phenylethylamine; VMA, vanillylmandelic acid; NE, norepinephrine.

**References**

1. Mao Q, Gong X, Zhou C, Tu Z, Zhao L, Wang L, Wang X, Sun L, Xia J, Lian B, Chen J, Mu J, Yang D, Xie P. Up-regulation of SIRT6 in the hippocampus induced rats with depression-like behavior via the block Akt/GSK3β signaling pathway. Behav Brain Res. 2017, 323:38-46.

2. Gong X, Huang C, Yang X, Mao Q, Zeng L, Zheng P, Pu J, Chen J, Wang H, Xu B, Zhou C, Xie P. Proteomic analysis of the intestine reveals SNARE-mediated immunoregulatory and amino acid absorption perturbations in a rat model of depression. Life Sci. 2019, 234:116778.

3. Wu M, Tian T, Mao Q, Zou T, Zhou CJ, Xie J, Chen JJ. Associations between disordered gut microbiota and changes of neurotransmitters and short-chain fatty acids in depressed mice. Transl Psychiatry, 2020, 10(1):350.

4. Tian Tian, Qiang Mao, Jing Xie, Ying Wang, Wei-hua Shao, Qi Zhong, Jian-jun Chen. Multi-omics data reveals the disturbance of glycerophospholipid metabolism caused by disordered gut microbiota in depressed mice. Journal of Advanced Research, 2021, doi.org/10.1016/j.jare.2021.10.002.

5. Zheng P, Zeng B, Zhou C, Liu M, Fang Z, Xu X, Zeng L, Chen J, Fan S, Du X, Zhang X, Yang D, Yang Y, Meng H, Li W, Melgiri ND, Licinio J, Wei H, Xie P. Gut microbiome remodeling induces depressive-like behaviors through a pathway mediated by the host's metabolism. Mol Psychiatry. 2016, 21(6):786-96.

6. Gong X, Huang C, Yang X, Chen J, Pu J, He Y, Xie P. Altered Fecal Metabolites and Colonic Glycerophospholipids Were Associated With Abnormal Composition of Gut Microbiota in a Depression Model of Mice. Front Neurosci. 2021, 15:701355.
